# Excitable dynamics of Ras triggers spontaneous symmetry breaking of PIP3 signaling in motile cells

**DOI:** 10.1242/jcs.224121

**Published:** 2019-03-04

**Authors:** Seiya Fukushima, Satomi Matsuoka, Masahiro Ueda

**Affiliations:** 1 Department of Biological Science, Graduate School of Science, Osaka University, Toyonaka, Osaka 560-0043, Japan; 2 RIKEN Center for Biosystems Dynamics Research (BDR), Suita, Osaka 565-0874, Japan; 3 Graduate School of Frontier Biosciences, Osaka University, Suita, Osaka 565-0871, Japan

**Keywords:** Cell signaling, Ras GTPase, Phosphatidylinositol lipid, Excitable system, Self-organization, Spontaneous migration

## Abstract

Spontaneous cell movement is underpinned by an asymmetric distribution of signaling molecules including small G proteins and phosphoinositides on the cell membrane. However, the molecular network necessary for spontaneous symmetry breaking has not been fully elucidated. Here, we report that, in *Dictyostelium discoideum*, the spatiotemporal dynamics of GTP bound Ras (Ras-GTP) breaks the symmetry due its intrinsic excitability even in the absence of extracellular spatial cues and downstream signaling activities. A stochastic excitation of local and transient Ras activation induced phosphatidylinositol (3,4,5)-trisphosphate (PIP3) accumulation via direct interaction with Phosphoinositide 3-kinase (PI3K), causing tightly coupled traveling waves that propagated along the membrane. Comprehensive phase analysis of the waves of Ras-GTP and PIP3 metabolism-related molecules revealed the network structure of the excitable system including positive-feedback regulation of Ras-GTP by the downstream PIP3. A mathematical model reconstituted a series of the observed symmetry-breaking phenomena, illustrating the essential involvement of Ras excitability in the cellular decision-making process.

## Abstract (precis)

**Summary:** The excitable dynamics of Ras GTPase breaks symmetry in signaling pathways to generate asymmetric signals, which are required for spontaneous cell migration.

## INTRODUCTION

Symmetry breaking underlies essential cellular decision-making processes, including polarization, migration and division. In eukaryotic amoeboid cells, a local accumulation of intracellular signaling molecules breaks symmetry. These asymmetric signals generate cell motility through cytoskeletal rearrangement (Bray, 1992; Karsenti, 2008). Several studies have investigated the underlying signaling mechanisms in various chemotactic cells and found common features in the network structures (Artemenko et al., 2014; Devreotes et al., 2017; Ecke and Gerisch, 2017; Janetopoulos and Firtel, 2008; Jin, 2013; King and Insall, 2009; Parent and Devreotes, 1999; Rappel and Edelstein-Keshet, 2017; van Haastert et al., 2017). For example, multiple signaling molecules including phosphatidylinositol lipids, Ras GTPases and various kinases accumulate locally on the cell periphery (Devreotes et al., 2017; King and Insall, 2009). These asymmetric signals occur even in the absence of a functional actin cytoskeleton or external asymmetry in chemoattractant stimulations (Arai et al., 2010; Funamoto et al., 2002; Parent et al., 1998; Sasaki et al., 2007). Therefore, the signaling network itself has the ability to exhibit symmetry breaking as internal spontaneous dynamics. However, it remains to be fully elucidated how the asymmetric signal emerges from the intracellular signaling network.

Recent evidence demonstrates that asymmetric signals can spontaneously arise from the excitability of the signaling network (Huang et al., 2013; Nishikawa et al., 2014; Shibata et al., 2012). Excitability in biology has been well documented experimentally and theoretically in the action potentials of neurons (Hodgkin and Huxley, 1952; Krahe and Gabbiani, 2004). Similar findings have since been made in many other cellular phenomena, such as cell differentiation (Eldar and Elowitz, 2010; Norman et al., 2015), gene expression (Loewer et al., 2010; Mönke et al., 2017) and eukaryotic chemotaxis (Shi and Iglesias, 2013; Xiong et al., 2010). In general, an excitable system has a threshold for all-or-none excitation, ensuring that cells can respond only to a supra-threshold stimulus in an all-or-none manner, although some cells exhibit spontaneous excitation due to internal fluctuations or molecular noise in the absence of an external stimulus. In addition, a refractory period follows each excitation in which the system is unable to respond to the stimulus. Excitable systems set under supra-threshold conditions exhibit oscillations or traveling waves (Lindner et al., 2004). These features require two feedback mechanisms: one is positive-feedback regulation for the all-or-none response to a supra-threshold stimulus, and the other is delayed negative-feedback for the temporal decline of the response followed by the refractory period. Finally, a signaling domain is generated locally on the cell membrane to induce symmetry breaking.

Chemotactic signaling pathways in *Dictyostelium discoideum* exhibit the characteristics of excitable systems (Nishikawa et al., 2014; Shibata et al., 2012; Tang et al., 2014). The phosphatidylinositol (3,4,5)-trisphosphate (PIP3), TorC2, PLA2 and soluble guanylate cyclase (sGC) pathways located downstream of chemoattractant receptors can, in parallel, each generate an intracellular cue for symmetry breaking during cell migration (Chen et al., 2007; Funamoto et al., 2002; Iijima and Devreotes, 2002; Kamimura et al., 2010; Parent et al., 1998; Tanabe et al., 2018; Veltman et al., 2008). The chemoattractant gradient signals are mediated by G-protein-coupled receptors, heterotrimeric G proteins and Ras GTPases, and bias the asymmetric signals along the gradient direction for chemotaxis (Devreotes et al., 2017). In the PIP3 pathway, the PIP3-enriched domain acts as the asymmetric signal on the cell membrane at the front (Huang et al., 2013; Weiger et al., 2009). Evidence for excitability in the PIP3 pathway includes stimulation-induced all-or-none excitation, refractory behavior, spontaneous excitation and traveling wave generation (Knoch et al., 2014; Miao et al., 2017; Nishikawa et al., 2014; Shibata et al., 2012). Traveling waves of the PIP3-enriched domain have been seen in living *Dictyostelium* cells and can be explained by various mathematical models (Shibata et al., 2013; Xiong et al., 2010). On the other hand, it has long been well known that chemoattractant gradients often induce stationary PIP3-enriched domains facing the chemoattractant source in *Dictyostelium* cells, but this phenomenon has not been reconstituted theoretically (Janetopoulos et al., 2004; Parent and Devreotes, 1999; Sasaki et al., 2004; Shibata et al., 2013; Wang et al., 2013; Xu et al., 2007). Consistent with this, the molecular network configuration that explains these apparently contradicting observations has not been elucidated. In addition to the excitable dynamics, recent reports have revealed that the bistable dynamics of PIP3 is generated through mutual inhibition between PIP3 and PTEN and this mutual inhibition exists between other molecules in polarized cells (Li et al., 2018; Matsuoka and Ueda, 2018). The bistable system can produce two stable states (i.e. PIP3-enriched and PIP3-depleted states) and does not necessarily oscillate, providing a basis for the stationary dynamics of the PIP3-enriched domain.

Here, we performed quantitative live-cell imaging analysis to reveal the spatiotemporal relationship between several major signaling components, including Ras-GTP, PI3K, PIP3 and PTEN. We found Ras-GTP is central for the emergence of excitable dynamics independently of upstream chemoattractant sensing or downstream parallel signaling pathways. The network configuration study suggests that there is coupling between the excitable Ras network and a bistable PIP3/PTEN network via PI3K. Feedback regulation of the Ras excitability from downstream PIP3 stabilized the asymmetric signal, suggesting signal integration occurs at the level of excitable Ras dynamics to modulate cell motility. A reaction–diffusion model reproduced these experimental results successfully, illustrating the central role of Ras excitability in spontaneous symmetry breaking during cell migration.

## RESULTS

### Ras wave formation is independent of PIP3 and other downstream pathways

**Fig. 1.**
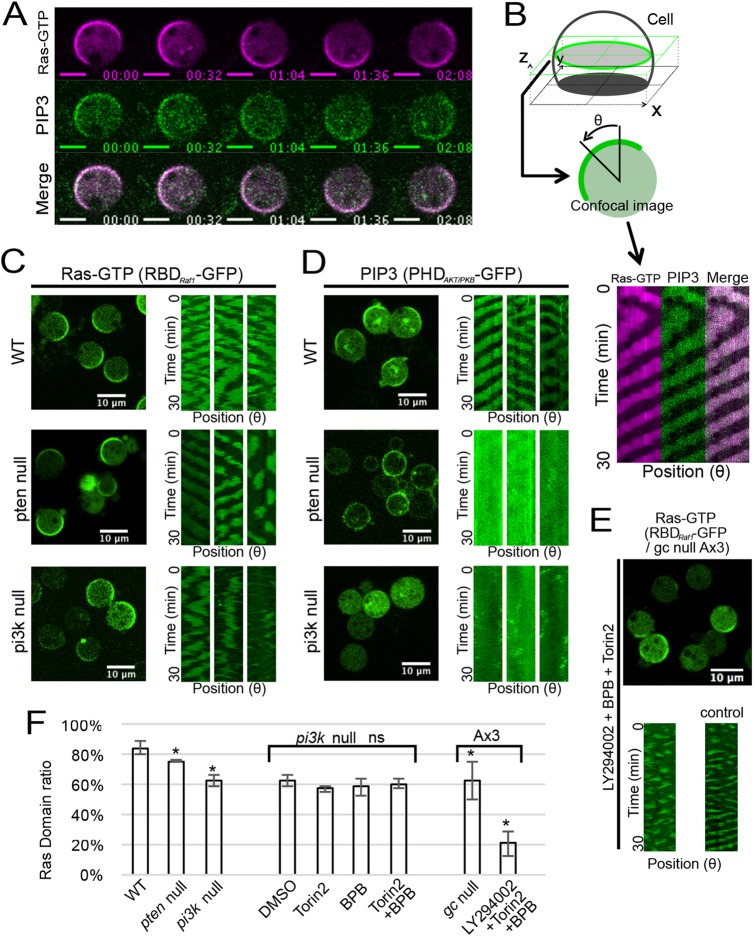
**Ras waves in the absence of active downstream parallel pathways.** (A) Simultaneous time-lapse of Ras-GTP and PIP3 waves in WT cells expressing RBD_Raf1_–RFP and PHD_AKT/PKB_–GFP taken by confocal microscopy. Scale bars: 5 µm. Time format is ‘mm:ss’. (B) Kymograph analysis of images as in A. (C,D) Confocal images (left) and typical kymographs (right) of Ras and PIP3 waves in WT, *pten*-null and *pi3k1-5*-null cells. PIP3 waves were not observed in *pten*-null or *pi3k1-5*-null cells. (E) Confocal image and kymographs of *gc*-null cells (Ax3 strain) treated with a combination of 100 µM LY294002, 10 µM Torin2 and 2 µM BPB. (F) Ratio of the number of cells showing Ras-GTP-enriched domains. Data are mean±s.d. of three independent experiments, and more than 200 cells were counted in each experiment. *P<0.01, Welch's *t*-test against WT; ns, not significant, *P*>0.01, Welch's *t*-test against *pi3k1-5* null.

We performed live-cell imaging analysis of both Ras-GTP and PIP3 by using RBD_Raf1_–GFP (or RFP) and PHD_AKT/PKB_–GFP, two fluorescent reporters specific for Ras-GTP and PIP3, respectively (Sasaki et al., 2004). To avoid effects mediated by the actin cytoskeleton in the Ras-GTP and PIP3 dynamics, the cells were treated with the actin polymerization inhibitor latrunculin A. Following the method described previously (Arai et al., 2010), the cells were also treated with 4 mM caffeine to observe waves traveling along the membrane. Under confocal microscope observation, Ras-GTP and PIP3 exhibited traveling waves along the cell periphery in *Dictyostelium* cells treated with both latrunculin A and caffeine (Fig. 1A; Movie 1), consistent with previous observations (Miao et al., 2017; Shibata et al., 2012; van Haastert et al., 2017). A kymograph showing the intensities of both probes along the membrane clearly indicated colocalizing Ras and PIP3 waves in the background of wild-type (WT) cells (Fig. 1B).

To see whether the generation of the traveling wave of Ras-GTP requires the PIP3 wave, we observed both probes in *pi3k1-5*-null (a strain that is null for the five PI3Ks PI3K1, PI3K2, PI3K3, PI3K4 and PI3K5) and *pten*-null strains, because PI3K and PTEN are essential for the production and degradation of PIP3, respectively (Comer and Parent, 2002; Funamoto et al., 2001; Iijima and Devreotes, 2002). Ras-GTP exhibited wave propagation even without PI3K or PTEN, whereas PIP3 did not (Fig. 1C,D). The efficiencies of Ras wave generation in WT, *pi3k1-5* and *pten*-null cells were 84% (*n*=1541), 63% (*n*=1487) and 76% (*n*=1982), respectively (Fig. 1F), indicating PIP3 production and degradation are not necessary for Ras wave generation. We noticed that Ras-GTP waves exhibited zigzag or disconnected patterns with prolonged oscillatory periods in both mutants compared with WT (Fig. S1C), suggesting a partial contribution of PIP3 production and PIP3 degradation to the maintenance of Ras waves.

We further examined the possible involvement of other parallel chemotactic signaling pathways in the generation of the Ras wave. When we applied Torin2 and BPB, which are inhibitors of the TorC2 and PLA2 pathways, respectively, to *pi3k1-5*-null cells, no obvious changes in Ras waves were observed (Fig. 1F; Fig. S1B). The efficiency of Ras wave generation in *gc*-null (*gca/sgca*-null) cells was reduced to a similar extent to that in *pi3k1-5*-null cells, suggesting a partial contribution of the sGC pathway along with the PIP3 pathway (Fig. 1F). Even in the cells with all major four pathways (the PIP3, TorC2, PLA2 and sGC pathways) inhibited, Ras waves were still observed, but with some defects (Fig. 1E,F; Fig. S1A) (Kortholt et al., 2011; Veltman et al., 2008). Thus, none of the four major pathways that mediate chemotactic signals downstream of Ras-GTP were necessary for Ras wave formation, although they may partially contribute to Ras activity via feedback loops. That is, the spatiotemporal dynamics of Ras GTPase is excitable without the activities of the downstream pathways.

### Ras waves trigger PIP3 waves via Ras-GTP–PI3K interaction

**Fig. 2.**
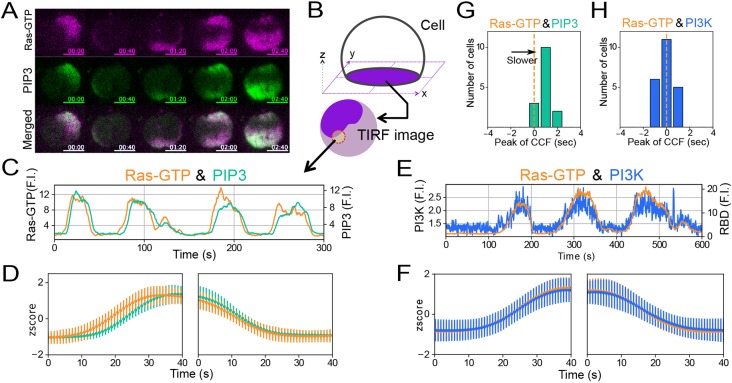
**Synchronized propagation of Ras, PIP3 and PI3K waves.** (A) Simultaneous time-lapse imaging of Ras-GTP and PIP3 taken by TIRFM. Scale bars: 5 µm. Time format is ‘mm:ss’. (B) Method of time trajectory analysis. (C,E) Typical examples of time trajectories of Ras-GTP (orange), PIP3 (green) and PI3K (blue). Fluorescence intensity (F.I.) was normalized to the minimum in each trajectory. (D,F) Average dynamics of the increasing (left) and decreasing phase (right). Orange, green and blue lines indicate Ras-GTP, PIP3 and PI3K, respectively. Data are the mean±s.d. from 15 cells and 22 cells, respectively. (G,H) Distribution of peak times of the cross-correlation functions (CCF). Dotted lines indicate time zero. The mean±s.d. peak value of Ras-GTP against PIP3 is 0.9±0.6 s (*n*=15 cells) and against PI3K is 0.0±0.7 s (*n*=22 cells).

In order to elucidate the temporal order between Ras-GTP and PIP3 stochastic traveling waves, we adopted a statistical method to analyze their average dynamics from a number of fluorescence time trajectories measured on the plasma membrane at a high signal-to-noise ratio through total internal reflection fluorescence microscopy (TIRFM) (Gerisch et al., 2011, 2012; Lange et al., 2016; Yang et al., 2015). Simultaneous observations of RBD_Raf1_–RFP and PHD_AKT/PKB_–GFP revealed that both probes exhibited closely coupled wave propagation on the membrane (Fig. 2A; Movie 2). We obtained time trajectories of the fluorescence intensities in a region of interest (ROI), and found that the PIP3 wave propagation was slightly delayed compared with the Ras wave propagation (Fig. 2B,C), which was obvious in the average dynamics obtained from 90 individual trajectories from 15 cells (Fig. 2D). We calculated the peak time of the cross-correlation function in each cell and obtained the distribution (Fig. 2G; Fig. S2C), which showed the lag time of PIP3 against Ras-GTP was ∼0.9±0.6 s on average.

We next analyzed the temporal relationship between Ras-GTP and PI3K in the wave propagation. We focused on PI3K2, which makes a significant contribution to the catalytic activity for PIP3 production and cell migration as a major effector of activated RasG among the six PI3Ks in *Dictyostelium discoideum* (Funamoto et al., 2002; Takeda et al., 2007). The localization of PI3K2 to the pseudopods of migrating cells depends on F-actin (Funamoto et al., 2002). We successfully visualized the wave dynamics of PI3K2 on the membrane of latrunculin A-treated cells by TIRFM observation (Fig. S2A). The oscillatory dynamics coincided tightly with that of Ras-GTP (Fig. 2E,F; Fig. S2B, Movie 3). The peak time of the cross-correlation function was 0.0±0.7 s on average (Fig. 2H; Fig. S2D), indicating no delay between the Ras-GTP and PI3K2 waves. When PI3K2–Halo-TMR (tetramethylrhodamine) and PHD_AKT/PKB_–GFP were observed simultaneously (Fig. S2E–I; Movie 4), the lag time of PIP3 against PI3K2 was ∼2.3±1.1 s on average, confirming the PIP3 waves follow the Ras and PI3K2 waves. The lag time of PIP3 against PI3K2 was longer than that of PIP3 against Ras-GTP, possibly due to the longer wave period in PI3K2–Halo- and PHD_PKB/AKT_–GFP-expressing cells than in RBD_Raf1_–RFP- and PHD_PKB/AKT_–GFP-expressing cells.

**Fig. 3.**
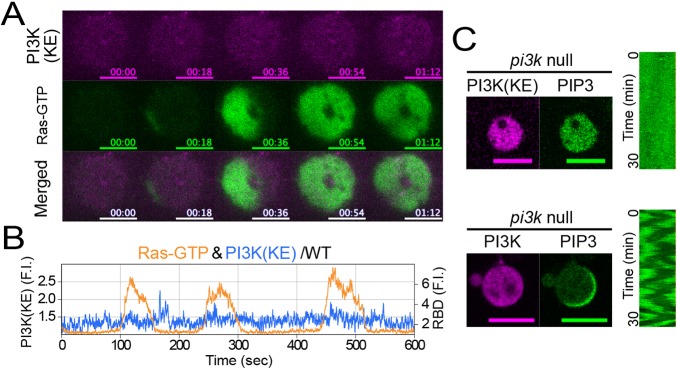
**Ras triggers PIP3 waves by regulating membrane translocation and PI3K activation.** (A) Simultaneous time-lapse imaging of RBD_Raf1_–GFP and PI3K2^K857E,K858E^–Halo-TMR taken by TIRFM. Scale bars: 5 µm. Time format is ‘mm:ss’. (B) Typical examples of time trajectories of PI3K^K857,858E^ (blue) and Ras-GTP (orange). (C) Confocal images of the rescue experiments for the *pi3k1-5*-null strain with PI3K and PI3K^K857E,K858E^. More than 100 cells were observed for each experiment. Scale bars: 10 μm.

We hypothesized that the F-actin-independent localization of PI3K2 to the membrane is due to interaction with Ras-GTP. To confirm this hypothesis, we used a PI3K2 mutant, PI3K2^K857E,K858E^, that is defective in Ras binding (Funamoto et al., 2002). PI3K2^K857E,K858E^ was distributed uniformly and was only present at low levels on the membrane with no wave generation during the Ras wave propagation in WT cells (Fig. 3A,B; Movie 5), indicating that the PI3K2 wave depends on interaction with Ras-GTP. Furthermore, we confirmed that the interaction between Ras-GTP and PI3K2 is required for PIP3 wave generation by rescue experiments. *pi3k1-5*-null cells exhibited PIP3 localization when transformed with PI3K2–Halo but not with PI3K2^K857E,K858E^–Halo (Fig. 3C; Fig. S2K). These results together demonstrate that Ras-GTP excitation triggers PIP3 waves through Ras-GTP interaction with PI3K, and that PIP3 is subordinate to Ras-GTP and PI3K.

### Feedback stabilizes Ras waves in the default state

**Fig. 4.**
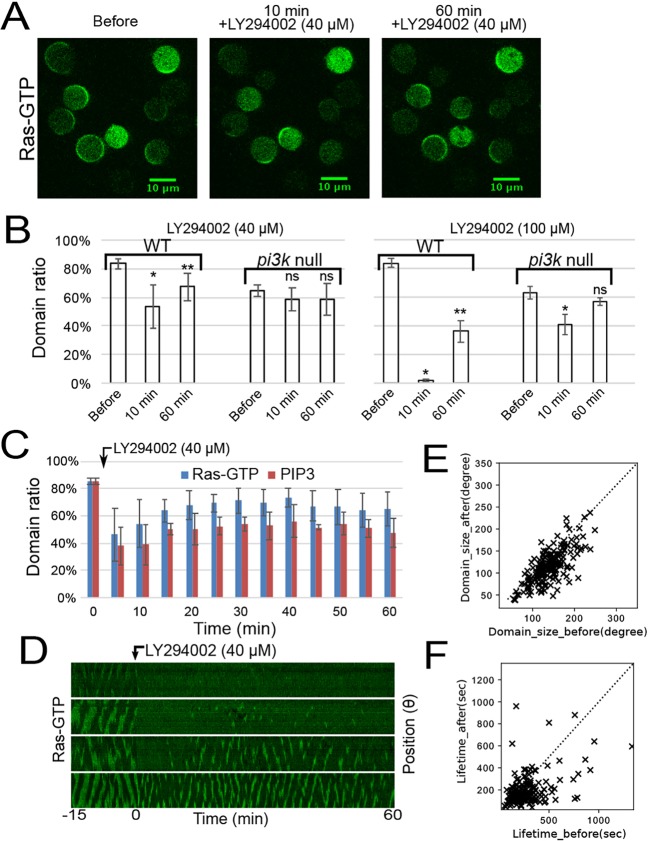
**The effect of PI3K inhibition on Ras waves.** (A) Response of Ras waves to a PI3K inhibitor (40 μM LY294002) before (left) and after 10 min (center) or 60 min (right) of treatment. (B) Ratio of the number of cells showing Ras-GTP domains before and after treatment with 40 µM or 100 µM LY294002 in WT and *pi3k1-5*-null strains. Data are mean±s.d. of three independent experiments. More than 45 cells were counted in each experiment. *P<0.01, **P<0.01, ns, not significant, Welch's *t*-test; comparisons were made with the ‘Before’ column for the same cell type. (C) Ratio of the number of cells showing Ras-GTP or PIP3 domains after treatment with LY294002. Data are mean±s.d. of three independent experiments. More than 54 RBD_Raf1_–GFP and PHD_AKT/PKB_–RFP doubly expressing cells were observed. (D) The kymographs show typical responses of Ras waves before and after 40 µM LY294002 treatment. (E,F) Distribution of the domain size or lifetime transition of the Ras wave pattern in each cell. Dotted lines indicate where Lifetime (before) equals Lifetime (after). These values were measured and averaged in each cell; 191 recovered cells were measured.

In contrast to Ras waves being generated in the *pi3k1-5*-null strain (Fig. 1), previous studies have shown that Ras waves are suppressed by the PI3K inhibitor LY294002 (Arai et al., 2010; Loovers et al., 2006). We investigated this apparent inconsistency between the genetic and pharmacological inhibition. When treated with 40 µM LY294002, Ras-GTP waves vanished in cells within 10 min, as reported previously, but those in *pi3k1-5*-null cells were relatively unaffected (Fig. 4B, left; Fig. S3B,E), suggesting that PI3K and PIP3 positively regulate Ras-GTP waves by constituting a positive-feedback loop. However, with prolonged observation, we found that the Ras waves recovered gradually in WT cells (Fig. 4A–D; Movie 8). Simultaneous observation of Ras-GTP and PIP3 revealed both waves disappeared from almost half of the cells in the population concomitantly, and that the Ras waves had recovered more effectively than the PIP3 waves by 60 min post treatment (Fig. 4C). In some cells, only Ras waves recovered, an effect more prominent with 100 µM LY294002 treatment, in which PIP3 waves were vanished in almost all cells (Fig. 4B, right; Fig. S3A,D). These results are consistent with our conclusion that Ras wave generation is essentially independent of PIP3 production, additionally suggesting a novel mechanism whereby Ras, PI3K and PIP3 waves are tightly coupled in WT cells at the default state. The recovery of Ras waves indicates that the system reaches another state without positive feedback, in which Ras regains excitability in some cells.

We noticed that the spatiotemporal patterns of Ras waves after LY294002 treatment were smaller and less continuous than those before treatment (Fig. 4D). Kymographs showed recovery of the Ras wave, although the Ras-GTP-enriched domains did not propagate continuously and instead underwent excitation transiently. The size and lifetime of the Ras-GTP-enriched domains became smaller and shorter, respectively, with LY294002 treatment (Fig. 4E,F). Similar defects in Ras waves were also observed in the *pi3k1-5*-null strain, as described above (Fig. S1C), but LY294002 treatment did not affect the size or lifetime of the domains in the *pi3k1-5*-null strain (Fig. S3B,C,E–G). Thus, PI3K activity is not essential for the generation of activated Ras-GTP-enriched domains, but feedback from PIP3 to Ras enhances the excitability to stabilize the asymmetric signals.

### Interrelationship analysis of traveling waves in the Ras–PIP3 system

**Fig. 5.**
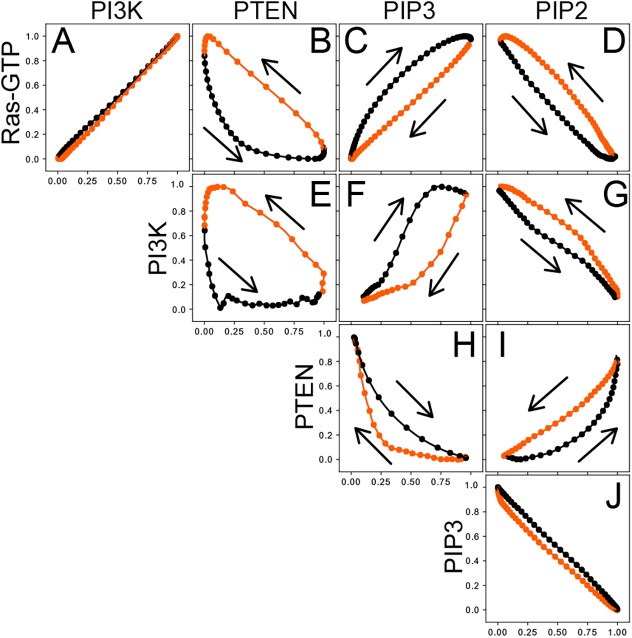
**Interrelationship between traveling waves of Ras-GTP, PI3K, PTEN, PIP3 and PIP2.** (A–J) Average dynamics of two arbitrary components (Ras-GTP, PI3K, PTEN, PIP3 or PIP2) are shown as an orbit in the corresponding coordinates. Arrows indicate temporal progression. Black lines indicate increased phase along the horizontal axis, and red lines indicate the decreased phase. Values are the normalized fluorescence intensities measured from the TIRFM images (Fig. 2D,F; Figs S2H, S4 and S5).

To reveal all intermolecular relationships relevant to Ras and PIP3 waves, we systematically analyzed the interrelationships between traveling waves of all possible combinations of two components among Ras-GTP, PI3K2, PTEN, PIP2 and PIP3. A fluorescent probe for PIP2, GFP-tagged Nodulin (GFP–Nodulin) was used, because it gave a brighter signal than the GFP-tagged PH domain derived from PLCδ1 (PHD_PLCδ1_–GFP) on the plasma membrane of *Dictyostelium* cells (Fig. S5, Movie 7) (Ghosh et al., 2015; Miao et al., 2017). We obtained phase diagrams from the average dynamics from simultaneous TIRFM observations (Fig. 5), which revealed that all these components exhibited traveling waves along the membrane in a closely coupled manner (Figs S4, S5, Movies 6, 7). We found five characteristic features in their relationships. First, a phase diagram in Ras-GTP–PI3K2 coordinates showed that the amount of PI3K2 on the membrane was proportional to that of Ras-GTP and tight regulation on PI3K membrane recruitment by Ras-GTP (Fig. 5A). The proportional relationship explains the resemblance in the phase diagrams for Ras-GTP–PTEN and PI3K2–PTEN (Fig. 5B–E), for Ras-GTP–PIP3 and PI3K2–PIP3 (Fig. 5C–F), and for Ras-GTP–PIP2 and PI3K2–PIP2 (Fig. 5D–G). Second, the Ras-GTP–PTEN and PI3K2–PTEN diagrams exhibited a triangle shape with counterclockwise time progression (Fig. 5B,E). In the initial moment of the decreasing phase of the Ras-GTP and PI3K2 waves, the decrease in the amount of Ras-GTP and PI3K2 on the membrane was followed by a delayed increase in PTEN, revealing an order in the temporal changes of these molecules. Third, the Ras-GTP–PIP3 and PI3K2–PIP3 diagrams showed a characteristic ellipse-like trace with temporal clockwise progression, which is consistent with Ras and PI3K2 waves preceding PIP3 waves (Fig. 5C,F). Fourth, the PIP3–PIP2 diagram exhibited an inverse relationship with almost the same trace in the increasing and decreasing phases, suggesting that the total amount of PIP2 and PIP3 was almost constant during the wave propagation (Fig. 5J; Fig. S5D, Movie 7). The inverse relationship explains the mirror-image relationship found between the Ras-GTP–PIP3 and Ras-GTP–PIP2 diagrams (Fig. 5C,D), the PI3K2–PIP3 and PI3K2–PIP2 diagrams (Fig. 5F,G), and the PTEN–PIP3 and PTEN–PIP2 diagrams (Fig. 5H,I). Finally, the PIP3–PTEN and PIP2–PTEN diagrams exhibited a previously reported crescent shape trace with clockwise temporal progression, showing that the PTEN increase follows the PIP3 decrease and PIP2 increase (Fig. 5H,I) (Arai et al., 2010).

### Modeling and simulation of symmetry breaking in the Ras–PIP3 system

**Fig. 6.**
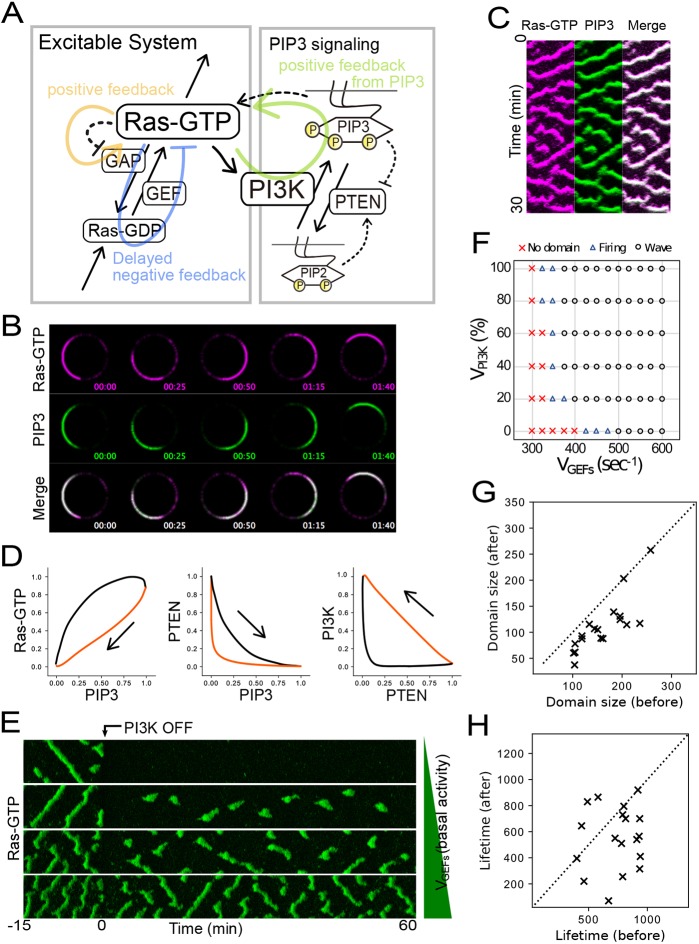
**Spatiotemporal simulation of Ras/PIP3 wave formation.** (A) Scheme of the Ras and PIP3 wave model used in this study. See text and Materials and Methods for details. (B) Ras-GTP and PIP3 traveling waves generated from the spatiotemporal stochastic simulation of the model shown in Fig. 6A. *V*_GEFs_=550 s^−1^. (C) Kymographs based on Fig. 6B. (D) Phase diagrams generated from the deterministic simulation (see also Fig. S6). (E) Spatiotemporal stochastic simulation of the model and PI3K inhibition. Kymographs show waves before and after PI3K inhibition. After PI3K inhibition, Ras waves vanished immediately, but recovered after a few minutes depending on the basal activity (*V*_GEFs_=400, 420, 450 and 500 s^−1^ from top to bottom). (F) Relationship between the Ras localization pattern and parameters (*V*_GEFs_= 300–600 s^−1^ in 25 increments and V_PI3K_=0–100% in 20% increments). (G,H) Distribution of the domain size and lifetime transition of the Ras wave pattern in each cell. Dotted lines indicate where Lifetime (before) equals Lifetime (after) (*V*_GEFs_=400–570 s^−1^ in 10 increments).

The dynamics of the components can be naturally explained and reconstituted in a numerical simulation by using a model described by a series of reaction–diffusion equations based on the experimental observations (Fig. 6A, see Materials and Methods for detail). Ras excitability was modeled with positive and delayed negative feedbacks on Ras, two features commonly assumed in excitable systems and adopted in previous models for the self-organization of PIP3-enriched domains (Arai et al., 2010; Knoch et al., 2014; Xiong et al., 2010). PI3K on the membrane is proportional to Ras-GTP levels and catalyzes PIP3 production according to a simple Michaelis–Menten (MM)-type enzymatic reaction. PTEN is recruited to the membrane via interaction with PIP2 and excluded from the membrane by interaction with PIP3, phenomena that are also described by MM-type binding reactions (Arai et al., 2010; Iijima et al., 2004; Matsuoka and Ueda, 2018; Matsuoka et al., 2013). The observed feedback was modeled so that an assumptive regulator activates Ras in a PIP3-dependent manner (Fig. 4). To reconstitute the inverse linear relationship between PIP3 and PIP2 (Fig. 5; Fig. S5), no other reaction pathways that metabolize PIP3 and PIP2 were considered in our model.

We performed a stochastic simulation and confirmed that the model can reconstruct all the traveling waves in a manner consistent with the experimental observations (Fig. 6B,C). By assuming no PI3K activity, the one-dimensional spatiotemporal simulation reconstructed Ras-GTP traveling waves without PIP3 waves due to the excitability (Fig. S6A). By applying PI3K activity, the traveling waves of PIP3 also appeared in a colocalized manner with Ras-GTP (Fig. 6B,C). Moreover, the wave period became shorter due to positive feedback from PIP3 to Ras (Fig. S6A), which is consistent with the experimental result (Fig. S1C). Under this situation, the temporal oscillations of Ras-GTP, PI3K, PTEN, PIP2 and PIP3 were all reconstructed, and phase diagrams plotted in the corresponding coordinates were consistent with the experimental observations showing the five characteristic features in their relationships (Fig. 6D; Fig. S6B). Thus, the interrelated oscillations of the Ras-GTP–PIP3 system can be explained by our model, in which activated Ras triggers the other waves through its interaction with PI3K. Since the PIP3 and PTEN system provides bistability of the PIP3-enriched/PTEN-depleted and PIP3-depleted/PTEN-enriched states (Matsuoka and Ueda, 2018), the PIP3–PTEN bistable state is subordinate to the Ras-GTP excitable state due to the Ras-GTP–PI3K interactions, providing traveling wave coupling.

We next examined the effects of positive feedback from PIP3 on Ras wave generation by turning on/off PI3K activity under broader conditions of the simulation. We performed the simulation by changing the *V*_GEFs_ value, which determines the basal activity of guanine nucleotide exchange factors (GEFs) that act independently of PIP3 signaling. In the presence of PI3K activity, that is, positive feedback from PIP3 to Ras, traveling waves were generated across a wide range of *V*_GEFs_ (Fig. 6E,F). Upon the inhibition of PI3K and thus no feedback regulation, Ras wave dynamics became dependent on *V*_GEFs_. Under intermediate conditions, Ras waves recovered after a transient loss. The transient loss and recovery can be explained as follows. In our model, a larger *V*_GEFs_ causes higher Ras-GTP basal levels and thereby a smaller distance to the threshold in the excitable system. At an intermediate *V*_GEFs_, PI3K inhibition puts the system below the threshold, leading to a loss of Ras excitation. Then, the delayed negative feedback working on Ras gradually loses its suppressive activity, allowing the system to exceed the threshold and recover Ras excitation. During the recovery process, molecular noise helps stochastic crossing of the threshold, since the recovery was not reconstructed in deterministic simulations (Fig. S6C). The Ras excitation recovery can be also reproduced by increasing *V*_GEFs_ with a lag time after PI3K inhibition, an effect that can be regarded as changing the expression levels of the components responsible for Ras excitation. Furthermore, we performed kymograph analysis of the Ras-GTP dynamics obtained from the simulation and found that the size and lifetime of the Ras-GTP domain became smaller and shorter after the inhibition of PI3K activity, respectively, which is in good agreement with the experimental observations (Fig. 6G,H). These results suggest that feedback from downstream pathways contributes to the maintenance of Ras excitability.

## DISCUSSION

Live-cell imaging analysis of the asymmetric signal generation in the Ras and the PIP3 signaling pathway demonstrates that at least three characteristics of the self-organization process exist. First, Ras GTPase has a central role in the asymmetric signal generation to initiate traveling waves independently of downstream pathways including the PIP3 signaling pathway (Fig. 1). Second, Ras waves trigger the self-organization of the traveling waves of all components observed in the PIP3 signaling pathway through state transitions of the PIP3 and PTEN bistable system mediated by PI3K activation (Figs 2, 3 and 5). Third, feedback on Ras GTPase from the downstream molecules stabilizes the asymmetric signal generation (Fig. 4). Overall, these experimental observations demonstrate that Ras GTPase has excitable dynamics and governs spatiotemporal dynamics in the phosphatidylinositol lipid signaling pathway for asymmetric signal generation.

The PIP3-enriched domain exhibits both oscillatory and stationary dynamics. More specifically, it propagates along the cell membrane as traveling waves in the absence of the extracellular spatial cues, but stays at the same location facing the higher concentration in response to chemoattractant gradients (Janetopoulos et al., 2004; Parent and Devreotes, 1999; Sasaki et al., 2004; Wang et al., 2013; Xu et al., 2007). Current mathematical models are based on excitable systems, and thus they have succeeded in reconstituting the oscillatory dynamics but failed in reconstituting the stationary dynamics (Shibata et al., 2013; Xiong et al., 2010). Our study proposes a network configuration of the chemotactic signaling pathways that explain both the oscillatory and stationary dynamics. Ras-GTP constitutes an excitable network in the absence of downstream parallel PI3K, TorC2, GC and PLA2 pathways and of a functional actin cytoskeleton (Fig. 1). PIP3, PIP2, PI3K and PTEN constitute a bistable network based on the mutual inhibition between PIP3 and PTEN that generates PIP3-enriched/PTEN-depleted and PTEN-enriched/PIP3-depleted states (Matsuoka and Ueda, 2018). The bistable network, which cannot self-organize the traveling waves by itself, is subordinate to the excitable network such that direct interaction between Ras-GTP and PI3K couples these two networks to generate PIP3, PIP2, PI3K and PTEN waves in a synchronized manner with Ras waves in the absence of extracellular spatial cues (Fig. 3). In the presence of chemoattractant gradients, chemotactic signals can regulate the bistable network along with the excitable network via PTEN suppression, at least in part, by generating a stable PIP3-enriched domain that faces the chemoattractant source (Hoeller and Kay, 2007; Matsuoka and Ueda, 2018). By combining the excitable and bistable networks, our model can produce many chemotactic responses including (1) PIP3 signaling dynamics being free of oscillations when the relative contribution of the bistable network is strong and (2) dynamics exhibiting oscillations when the relative contribution of the excitable network is strong.

Our data show that the spatiotemporal regulation of Ras activity is a key event for the excitability and that functional actin cytoskeleton is not essential for triggering Ras excitation (Fig. 1A). Rather, the actin cytoskeleton can stabilize the Ras- and PIP3-enriched domains, since the domains are sometimes observed more stably at the leading edge of migrating *Dictyostelium* cells (Sasaki et al., 2007) than in latrunculin A-treated cells. The feedback regulation upon Ras and PIP3 from the actin cytoskeleton can contribute to the stable signaling and thus efficient cell migration, but the molecular mechanisms for the feedback regulation remain unknown. The excitable dynamics can be also observed in latrunculin A-washed out cells, in multinucleated giant cells and in PIP2-modulated cells without latrunculin A or caffeine treatment (Gerhardt et al., 2014; Gerisch et al., 2009; Miao et al., 2017). These experiments reveal whether the Ras signaling network works as a core of the excitable dynamics and how the actin cytoskeleton affects Ras activity. In addition, the morphological anterior–posterior polarity of *Dictyostelium* cells gradually becomes obvious with development after starvation, demonstrating that *Dictyostelium discoideum* is a good model for characterizing the polarity caused by the spatiotemporal dynamics of the excitable signaling network and cytoskeletal network.

Excitability requires fast positive and delayed negative feedback loops in the molecular network (Meinhardt, 1999). We have previously constructed a reaction–diffusion model for PIP3 waves in which the supply and degradation of PIP2 and PIP3 mediated by some enzymes other than PI3K and PTEN are assumed to constitute these feedback loops, causing an imbalance between PIP2 and PIP3 during the traveling wave propagation (Arai et al., 2010; Shibata et al., 2012). However, the inverse linear relationship between PIP2 and PIP3 shown here suggests these phosphoinositide species are balanced during the traveling wave propagation (Fig. 5; Fig. S5). Thus, terms describing the PI3K- and PTEN-independent supply and degradation of PIP2 and PIP3 can be excluded from the reaction–diffusion equation of the excitable network. In addition, the model that assumes PIP2, PIP3 and PTEN as constituents of the feedback loops for an excitable network is not realistic. Instead, we propose that PIP2, PIP3, PI3K and PTEN are subordinate to the excitability that arises from the GTP/GDP exchange on Ras. Although it is possible that PI(4,5)P2 affects Ras activity (Fets et al., 2014; Miao et al., 2017), our observations suggest that excitability does not require PIP3 and PIP2 metabolism under physiological conditions. Instead, multiple phosphoinositides, such as PI(3,4,5)P3, PI(4,5)P2 and PI(3,4)P2, might contribute to the mutual inhibition of polarized molecules (Li et al., 2018; Matsuoka and Ueda, 2018).

Ras GTPases and the regulatory network make up a complex system comprised of various Ras GTPase family proteins, and Ras GEFs and GTPase-activating proteins (GAPs). In *Dictyostelium discoideum* cells, at least 14 Ras GTPase family proteins, 25 GEFs and 14 GAPs are estimated from the genome sequence (Artemenko et al., 2014; Wilkins et al., 2005). Our findings here demonstrate that the Ras GTPase regulatory network has excitable dynamics, meaning that it has a threshold for excitation. In general, GEFs and GAPs regulate Ras GTPase positively and negatively, respectively. That is, the inputs from GEFs and GAPs can be regarded as excitatory and inhibitory signals for Ras GTPase. These signals are integrated through the regulation of Ras GTPase activity and cause excitation when the network exceeds the threshold. This concept is analogous to what occurs in a neuron in neuronal networks, because the neuron has characteristics of an excitable system in which excitatory and inhibitory signals derived from presynaptic cells are integrated in the post-synaptic cell during the signal processing. From the viewpoint of molecular signal processing in excitable systems, it is important to clarify the circuit structure of the Ras GTPase regulatory network in order to provide mechanistic insights into cellular decision-making processes. Because Ras and the phosphatidylinositol lipid signaling pathways are involved in oncogenesis and metastasis, understanding the mechanisms that regulate the threshold of the Ras excitable system are important issues in the biological and medical sciences.

## MATERIALS AND METHODS

### Cell culture and constructs

*Dictyostelium discoideum* wild-type Ax2 (our in-house strain) was used as the parental strain except when the *gc*-null strain (Ax3) was used (from the laboratory of Peter J. Van Haastert). Cells were transformed with plasmids based on the vector series pDM or pHK12 by electroporation. Plasmids were generated by Ligation or In-Fusion (TOYOBO, TaKaRa). All constructs were sequenced before transformation. To visualize the localization of PI3K2 and its mutants, Halo-tag^®^-fused PI3K2 was expressed and labeled with HaloTag^®^ TMR (tetramethylrhodamine) ligand. The activity of Halo-tag-fused PI3K2 was confirmed by rescue experiments on *pi3k1-5*-null mutants (Fig. 3C; Fig. S2J). A Ras-binding mutant of PI3K2 (PI3K2^K857E,K858E^) and truncations of PI3K2 N-terminal (PI3K2^Δ561-1859^) were obtained by PCR (TOYOBO). To observe the spatiotemporal dynamics of phosphatidylinositol lipids and their related enzymes, pairs of PHD_AKT/PKB_, Nodulin, PTEN, PI3K and RBD_Raf1_ tagged with GFP, RFP or Halo-TMR were co-expressed. Cells were cultured axenically in HL-5 medium containing G418 (20 mg/ml), Blasticydin S (10 mg/ml) or hygromycin (50 mg/ml) at 21°C (Watts and Ashworth, 1970).

### Preparation for live-cell imaging

For imaging experiments, cells were washed in 1 ml development buffer without Ca^2+^ or Mg^2+^ (DB−; 5 mM Na_2_HPO_4_ and 5 mM KH_2_PO_4_) twice and starved in 1 ml development buffer (DB; DB− with 2 mM MgSO_4_ and 0.2 mM CaCl_2_) for 3 to 4 h at a density of 5×10^6^ cells per ml on a 35 mm dish. The starvation time was optimized to observe Ras and PIP3 waves. Ras and PIP3 waves were observed in more than 80% of cells after a 3–4 h starvation without cAMP pulses. In this condition, cells were less polarized compared to starved cells subjected to repeated cAMP pulses (Fig. S1D) (Srinivasan et al., 2013). Halo-tag^®^-fused proteins were stained with 2 µM Halo-tag^®^ TMR ligand for 30 min and washed five times with 1 ml DB− before observation.

### Aggregation assay and development assay

The aggregation assay was performed on a plastic dish. Cultured cells were washed in 1 ml DB− twice and incubated in 1 ml DB at a density of 5×10^6^ cells per ml on a 35 mm dish. We observed aggregation of the cells after 24 h. The development assay was performed on an agar plate. Approximately 10 to 20 cells were inoculated on a 90-mm LP agar plate (0.5% lactose, 0.5% peptone, 1.5% agar) with bacteria (*E. coli* B/r). We observed development after 5–6 days.

### Imaging with confocal microscopy

Confocal imaging was performed using an inverted microscope (ECLIPSE Ti; Nikon) equipped with a confocal unit (CSU-W1; Yokogawa). Laser sources for 488 nm and 561 nm excitation light were solid-state CW lasers (OBIS 488NM X 50MW and OBIS 561NM X 50MW, respectively; Coherent). Time-lapse images were acquired through a 60× oil immersion objective lens (CFI Apo TIRF 60X Oil, N.A. 1.49; Nikon) with an EM-CCD camera (iXon3 897; Andor). Cells were transferred to a 35 mm Glass Base Dish (Grass 12 φ, 0.15–0.18 thick; IWAKI) and suspended in 200 µl DB with 4 mM caffeine and 5 µM latrunculin A (Sigma). Caffeine was added to induce Ras and PIP3 waves and to inhibit cAMP relay (Shibata et al., 2012). Inhibitors were added after 15 min treatment with caffeine and latrunculin A. Latrunculin A (2 mM), LY294002 (40 µM or 100 µM; Cayman), Torin2 (1 mM; TOCRIS) and BPB (10 mM; TCI Japan) in DMSO were diluted to the final concentration (DMSO≤1%). The effectiveness of LY294002 during the 60 min observation was confirmed by replacing the extracellular medium (Fig. S3I). Time-lapse images were obtained at 200 ms exposure for each channel at 2 s intervals (488 nm laser power was ∼50 µW, and 561 nm laser power was ∼150 µW).

### Imaging with TIRFM

TIRF imaging was performed using an inverted microscope equipped with a handmaid prism-less TIR system (Axelrod, 2003). Laser sources for 488 nm and 561 nm excitation light were solid-state CW lasers (SAPPHIRE 488-20 and Compass 561-20, respectively; Coherent). Lasers were guided to the back focal plane of the objective lens (CFI Apo TIRF 60X Oil, N.A. 1.49; Nikon) through a back port of the microscope. TIR and EPI illumination were switched by tilting the incident angle of the lasers. The separated images were passed through dual-band laser split filter sets (Di01-R488/561-25×36, Di02R561-25×36, FF01-525/45-25 and FF01-609/54-25; Semrock) and captured by two EM-CCD cameras (iXon3 897; Andor) equipped with a 4× intermediate magnification lenses (VM Lens C-4×; Nikon). Cells were transferred to a cover glass (25 mm radius, 0.12-0.17 thick; Matsunami) that was fixed on a chamber (Attofluor^®^ Cell Chamber; Molecular Probes). The cover glass was washed by sonication in 0.1 M KOH for 30 min and in 100% ethanol for 30 min twice in advance. Cells were treated with 4 mM caffeine and 5 µM latrunculin A in DB for 15 min. Time-lapse images were obtained at 100 ms exposure for 10 min (488 nm laser power was ∼20 µW, and 561 nm laser power was ∼20 µW). Time-lapse images were smoothed with a 1 s time window to reduce shot noise.

### Image processing and analysis

The adjustment of images from a dual camera was performed on software by using objective micrometer images as a standard. The average error of the position after adjustment was less than 66 nm^2^. Leakage from one channel to the other was calibrated as:

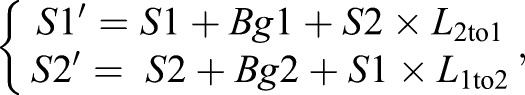
where *S*1 and *S*2 indicate the fluorescence signals from the two channels. *S′* indicates the detected signal, *Bg* indicates the background noise and *L* indicates the ratio of leakage from one signal to the other channel. Leakage parameters for GFP, RFP and TMR were obtained in advance.

The spatiotemporal dynamics were analyzed from the time trajectories of the membrane localization. For TIRF time-lapse images, time trajectories were the time-axis profiles of the average intensity of the ROI. We defined the ROI as a circle with a 0.2 µm (3 pixel) radius. In the case of confocal time-lapse images, the fluorescence intensities were analyzed as previously described (Arai et al., 2010). Fluorescence intensity along the rounded cell contours were measured in each frame. The intensity profiles were plotted against the angle θ and time as a 2D pattern. The line profile along the time axis was defined as the time trajectory of the membrane localization. This method can reduce the effect of stage drift.

### Time trajectory analysis

Raw trajectories were extracted from TIRF images as described above and designated as *F*(*t*). The auto-correlation function of *F*(*t*) and cross-correlation function of *F*_1_(*t*) and *F*_2_(*t*) for the first and second molecules observed in different colors were defined as:

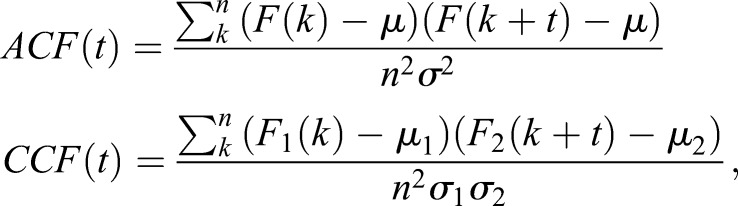
where μ and σ^2^ indicate the average and variance of *F*(*t*), respectively.

To obtain the average trajectories shown in Figs 2 and 3, we calculated the local maximums and minimums of the first derivative of the raw trajectories at the centers of the increasing and decreasing phases, respectively. Next, we extracted short trajectories of 60 s before and after the calculated centers from the raw trajectories. Finally, we aligned the short trajectories at their centers, normalized them to their *z*-scores and calculated the average trajectories. The phase diagrams were drawn from the average trajectories.

### Domain size and duration time analysis

The domain size and duration time were calculated from binarized kymographs. Kymographs were smoothed by applying a mean filter with a 9-pixel window and binarized to separate the domain and background. Then we detected each domain and defined the duration time as the size of the domain along the time axis and the domain size as the average spatial size of the domain in each time step. We ignored domains smaller than 32 s or shorter than 30 s.

### Reaction diffusion model and numerical simulations

To reconstruct the spatiotemporal dynamics of the Ras and PIP3 wave patterns, we constructed a combined model of the Ras and PIP3 signaling systems (Fig. 6A). As described in the main text, excitability and pattern formation derive from Ras and its regulatory network. The PIP3 signaling system follows Ras-GTP, and feedback from PIP3 to Ras maintains Ras excitability.

First, we constructed a model of the PIP3 signaling system as by the equations:
(1)
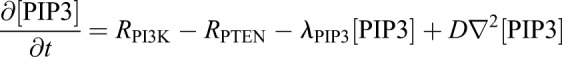

(2)
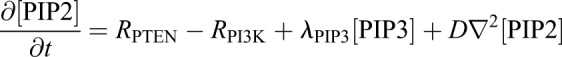

(3)
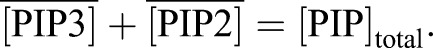


PIP3 is generated from PIP2 phosphorylation by PI3K (*R*_PI3K_) and dephosphorylated into PIP2 by PTEN (*R*_PTEN_) on the membrane (Eqn 1,2). The PTEN-independent PIP3 degradation rate, *λ*_PIP3_, is also introduced because the degradation of PIP3 is observed even in the *pten*-null strain (Iijima and Devreotes, 2002). The sum of average concentrations of PIP3 ([PIP3]) and PIP2 ([PIP2]) was made constant (3) (Fig. 5J). Therefore, we omitted PIP2 supply and PIP2 and PIP3 degradation from the model for simplicity. PIP3 and PIP2 diffuse on the membrane independently with the diffusion term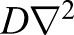.
(4)
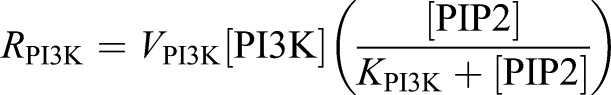

(5)
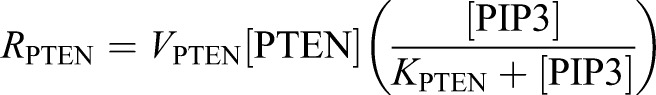

(6)
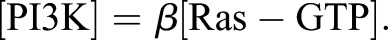


The PIP2 phosphorylation reaction (*R*_PI3K_) and PIP3 dephosphorylation reaction (*R*_PTEN_) were described as MM-type enzymatic reactions in which each term is composed of the maximum reaction rate (*V*_PI3K_, *V*_PTEN_) and Michaelis constant (*K*_PI3K_, *K*_PTEN_) (Eqn 4,5). Because PI3K shuttles between the cytosol and plasma membrane depending on Ras-GTP interactions, we introduced Ras-GTP-dependent PI3K membrane translocation and Ras-GTP-dependent PI3K activation (Eqn 6). *β* indicates the membrane association rate of PI3K, because the membrane translocation of PI3K is proportional to the Ras-GTP level (Fig. 5A).
(7)
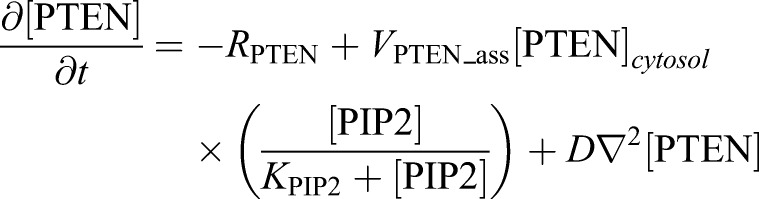

(8)
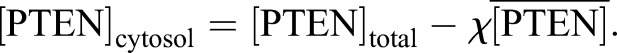


PTEN also shuttles between the cytosol and plasma membrane and shows a mutually exclusive localization pattern with PIP3 (Fig. 5H). To satisfy these constraints, we introduced PIP3-dependent exclusion of PTEN from the membrane (Arai et al., 2010; Iijima et al., 2004; Shibata et al., 2012). We assumed interaction between PTEN and PIP3 results in PTEN dissociation from the membrane and the dissociation rate has the same rate as the PIP3 dephosphorylation, *R*_PTEN_ (Eqn 7). Additionally, we introduced PTEN recruitment to the membrane by interaction with PIP2 (Iijima et al., 2004) as being dependent on the maximum reaction rate, *V*_PTEN_ass_, and Michaelis constant *K*_PIP2_ (Eqn 7). Unlike the case of PI3K, the cytosolic concentration of PTEN drastically changes before and after membrane translocation (Iijima and Devreotes, 2002). Therefore, we considered the cytosolic concentration of PTEN. As described previously, we assumed the cytosolic PTEN concentration is uniform inside the cytosol and described by Eqn 8 (Arai et al., 2010). Overline notation indicates average concentration, and *χ* indicates a constant that transforms the surface concentration on the membrane to a volume concentration in the cytosol.

Second, we constructed the Ras signaling regulation model as an excitable network. As a template for the excitable network model, we applied the theoretical model of self-organization on the membrane reported previously (Shibata et al., 2012), which can reconstruct well the excitable responses of a Ras/PIP3 signaling system with a minimum number of elements.
(9)
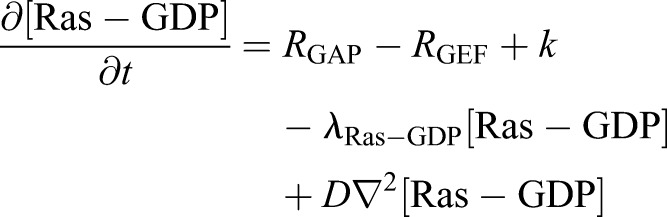

(10)
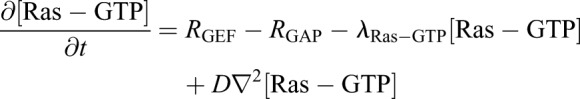

(11)
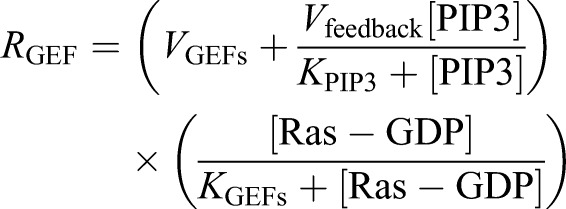

(12)
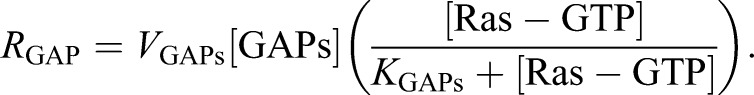


In this model, we introduced a positive regulator and negative regulator of Ras as GEFs and GAPs, respectively. Ras is activated by the reaction *R*_GEFs_ and inactivated by reaction *R*_GAPs_. Additionally, Ras-GDP is supplied and consumed by membrane association and dissociation at the rates *k* and *λ*_Ras-GDP_, respectively (Eqn 9). Ras-GTP dissociates from the membrane at *λ*_Ras-GTP_ and is supplied only by the GTP exchange reaction of Ras-GDP (Eqn 10). The GDP/GTP exchange reaction reduces the Ras-GDP level, which reduces the Ras-GTP supply.

R_GEFs_ is described as a MM-type enzymatic reaction composed of two terms (Eqn 11). One term defines the basal activity of Ras and the other defines feedback from PIP3. Each term is composed of the maximum reaction rate (*V*_GEFs_, *V*_feedback_) and Michaelis constant *K*_GEFs_. This formulation is based on the experimental observation that PIP3 production influences the formation of Ras waves (Fig. 4). R_GAPs_ is described as an enzymatic reaction with maximum reaction rate *V*_GAPs_ and Michaelis constant K_GAPs_ (Eqn 12).
(13)
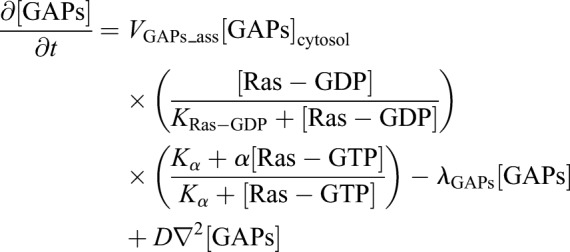

(14)
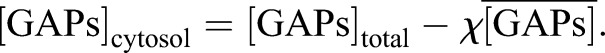


In order to promote the formation of a Ras-GTP localization patch, we introduced positive regulation from Ras-GDP and negative regulation from Ras-GTP on the recruitment of GAPs to the membrane. These regulations are composed of two positive-feedback loops and result in a mutually exclusive relationship between Ras-GTP and GAPs, which stabilizes spatially restricted Ras-GTP localization patches. Positive regulation from Ras-GDP is described as a MM-type enzymatic reaction composed of a maximum reaction rate (*V*_GAPs_ass_) and Michaelis constant (*K*_Ras-GDP_), and negative regulation is described following a previous study (Arai et al., 2010). Additionally, we introduced the degradation of GAPs at rate λ_GAPs_ and the cytosolic concentration as described in Eqn 13.

Detailed methods for the numerical simulations are described previously (Shibata et al., 2012). We evaluated a one-dimensional system with 100 grids along the membrane to reconstruct the results of the kymographs. The radius of the cells was chosen to be 5 µm with a constant time step of Δ*t*=0.005. For the stochastic simulation, we used Tau-leaping (Gillespie, 2001). The spatiotemporal dynamics are described by the reaction diffusion equations described above (Eqns 1–14).

The parameters used are summarized in Tables S1 and S2. First, we set the parameters shown in Table S1, which are related to the PIP3 signaling pathway. We performed a simulation in which the concentration term [Ras-GTP] was fixed to the value from the experiments, and adjusted the parameters to satisfy the relationships between PIP3, PI3K and PTEN to the experimental results shown in Fig. 5. Next, we set the parameters related to the Ras wave pattern formation in the condition without feedback from PIP3 by a simulation in which the feedback term *V*_feedback_ was fixed to zero. Finally, we modulated *V*_feedback_ and *V*_GEFs_ to satisfy the experimental results. The initial concentrations are summarized in Tables S1 and S2. The initial condition is spatially uniform, and it takes ∼3 to 5 min for the system to show wave patterns. In the case of the deterministic simulation, we used final values of the stochastic simulation as the initial condition.

Kymographs were generated from the simulations based on this model and parameters. The parameter value of PI3K activity, V_PI3K_, was modulated during the simulation. Then, we set the value of V_PI3K_ to 0 s^−1^ (PI3K off) or 12 s^−1^ (PI3K ON) in the PI3K on/off simulation shown in Fig. 6E. The value of Ras basal activity, V_GEFS_, was also changed in each simulation. We varied the values of *V*_GEFs_ from 400 to 600 s^−1^ in ten increments and analyzed all kymographs to obtain distributions of the domain size and duration time.

## Supplementary Material

Supplementary information
