# Supplementary material for: Excitable dynamics of Ras triggers spontaneous symmetry breaking of PIP3 signaling in motile cells
Source: J Cell Sci. 2019 Mar 4;132(5):jcs224121. doi: 10.1242/jcs.224121 (PMC6432713; doi:10.1242/jcs.224121)
Supplement: Supplementary information [file joces-132-224121-s1.pdf]

Supplementary information

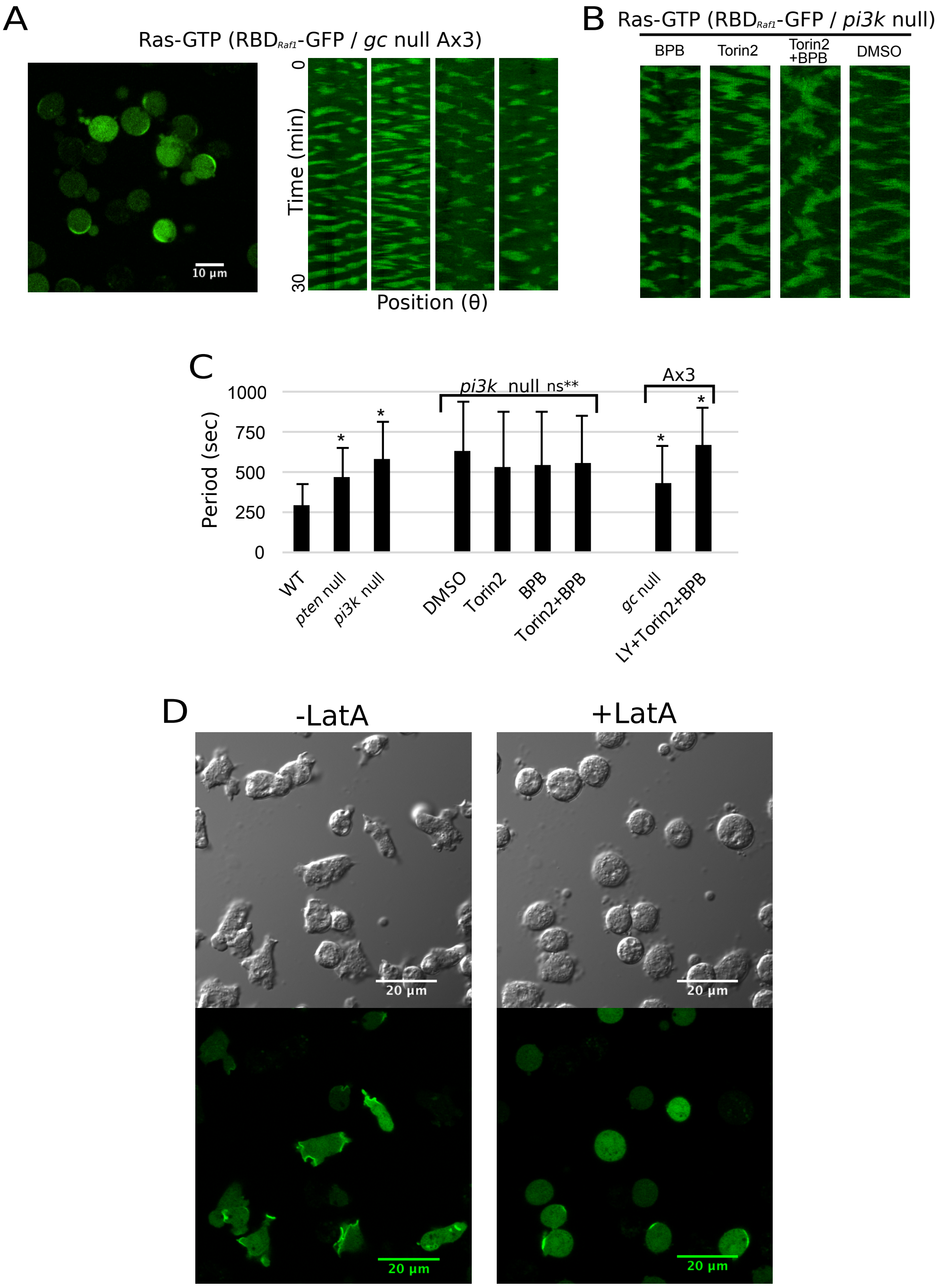

Fig. S1. Ras waves in mutants with LY294002 treatment. (A) Confocal images of Ras waves in AX3 background *gc* null cells expressing RBD_{Raf1}-GFP. Kymographs shows typical Ras wave patterns. (B) Kymographs of *pi3k1-5* null cells treated with 2 μM BPB, 10 μM Torin2, a combination of the two or 1% DMSO as a control. (C) Ras wave periods were calculated from the auto-correlation function of more than 20 cells and averaged. (* $P < 0.01$ Welch's t-test against WT; ns** $P > 0.01$ Welch's t-test against *pi3k1-5* null). (D) DIC image and RBD_{Raf1}-GFP fluorescent image of the cell before and after 5 μM Latrunculin A treatment.

Journal of Cell Science • Supplementary information

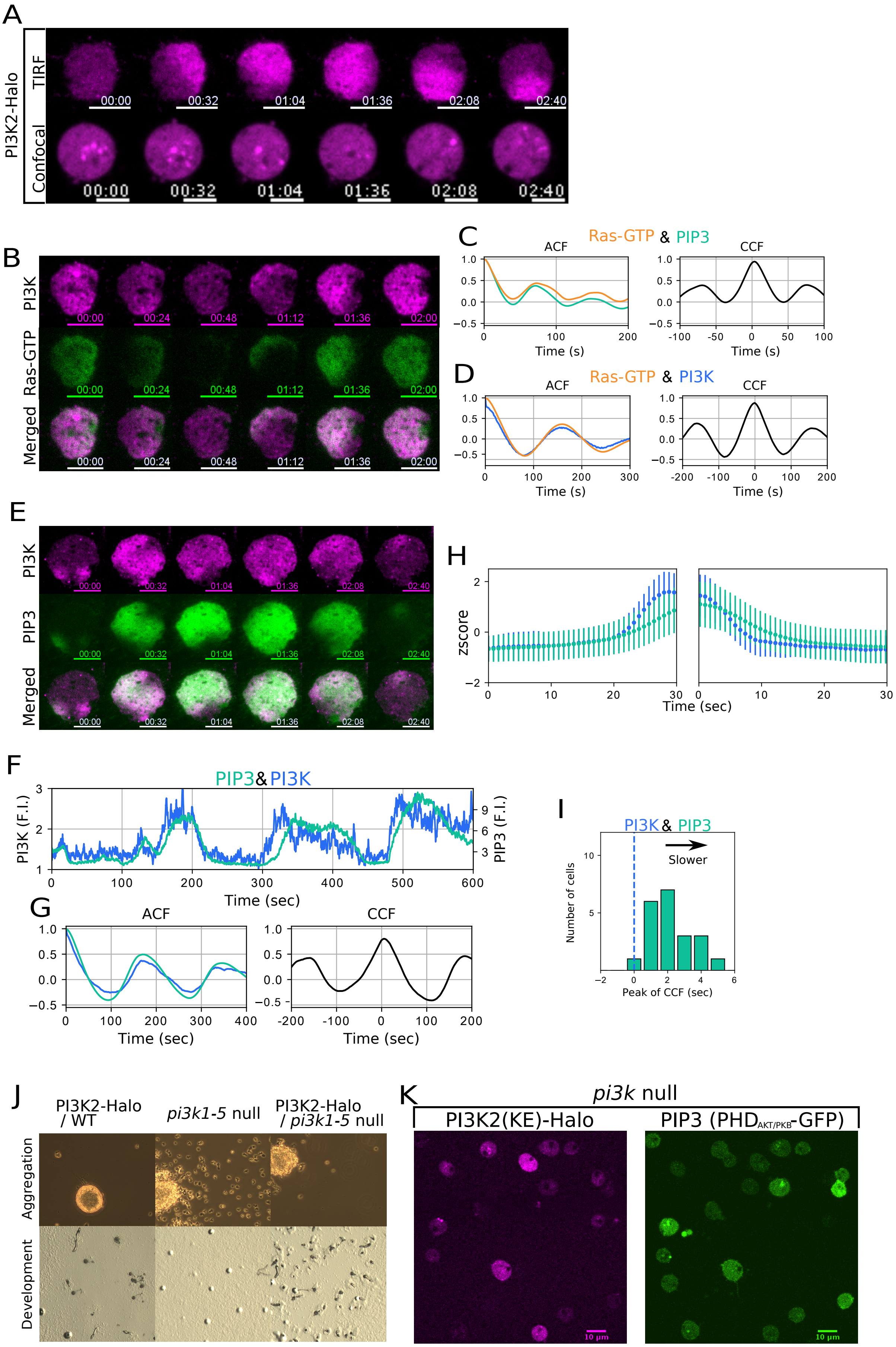

Journal of Cell Science • Supplementary information

Fig. S2. TIRF and confocal imaging of PI3K. (A) Time-lapse imaging of PI3K (PI3K2-Halo-TMR) taken by a TIRF microscope (top) and confocal microscope (bottom) in *pi3k1-5* null cells. Scale bars, 5 μm. (B) Simultaneous time-lapse imaging of RBD_{Raf1}-GFP and PI3K2-Halo-TMR taken by TIRFM. Scale bars, 5 μm. Time format is “mm:ss”. (C), (D) Auto- and cross-correlation functions of the trajectories shown in Fig. 2C and 2E, respectively. (E) Simultaneous time-lapse TIRFM imaging of PI3K2-Halo-TMR and $PHD_{AKT/PKB}$-GFP. (F) Typical examples of time trajectories of PI3K (blue) and PIP3 (green). (G) Auto- and cross-correlation functions of the trajectories shown in Fig. S2B. (H) Average trajectories of the increase phase (left) and decrease phase (right). Data are the mean ± s.d. from 21 cells. (I) Distribution of peak times of the cross-correlation functions. Dotted line indicates time zero. The average peak value is 2.3 ± 1.1 s (n = 21 cells). (J) Rescue experiments of aggregation and development of PI3K2-Halo-expressing *pi3k1-5* null cells. (K) Confocal images of *pi3k1-5* null strain expressing PI3K(KE)-Halo and $PHD_{AKT/PKB}$-GFP. PIP3 domain formation is not rescued in these cells.

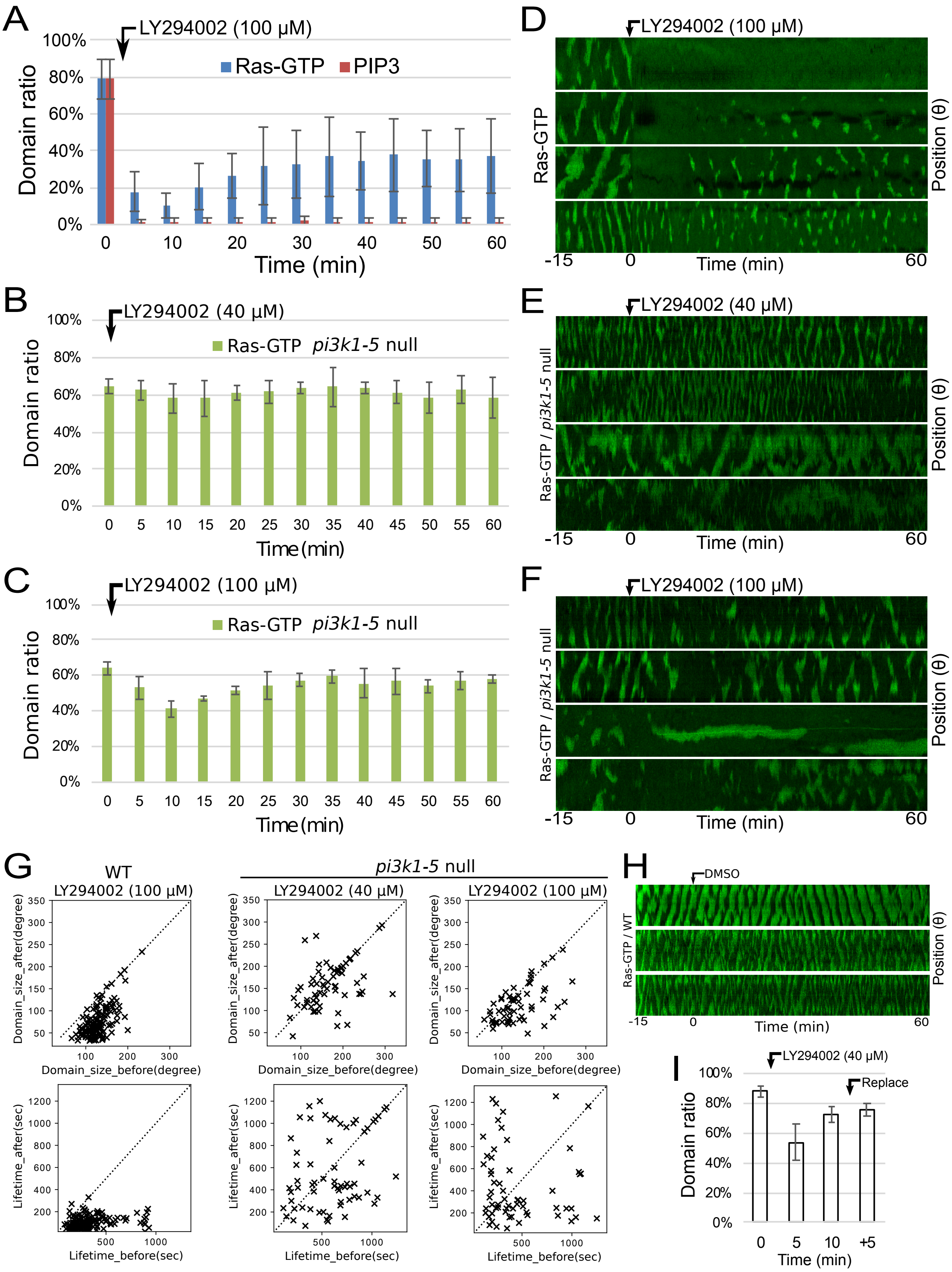

Journal of Cell Science • Supplementary information

Fig. S3. Ras waves with LY294002 treatment. (A) Ratio of cells showing Ras-GTP or PIP3 domains after treatment with 100μM LY294002 in WT cells. Data are mean ± s.d. of four independent experiments. More than 42 cells were counted in each experiment. (B, C) Ratio of the cells showing Ras-GTP domains after treatment with 40 or 100 μM LY294002 in *pi3k1-5* null cells. Data are mean ± s.d. of three independent experiments. More than 45 or 50 cells were counted in each experiment. (D-F) The kymograph shows a typical response of a Ras wave in response to LY294002 treatment. (G) Distribution of the domain size or lifetime transition of the Ras wave pattern in each cell. 140, 66 and 63 cells (left to right) were measured. (H) The kymograph shows Ras waves in WT cells treated with 0.25% DMSO. (I) Experiments in which the extracellular medium was replaced with fresh medium containing the same concentration of LY294002.

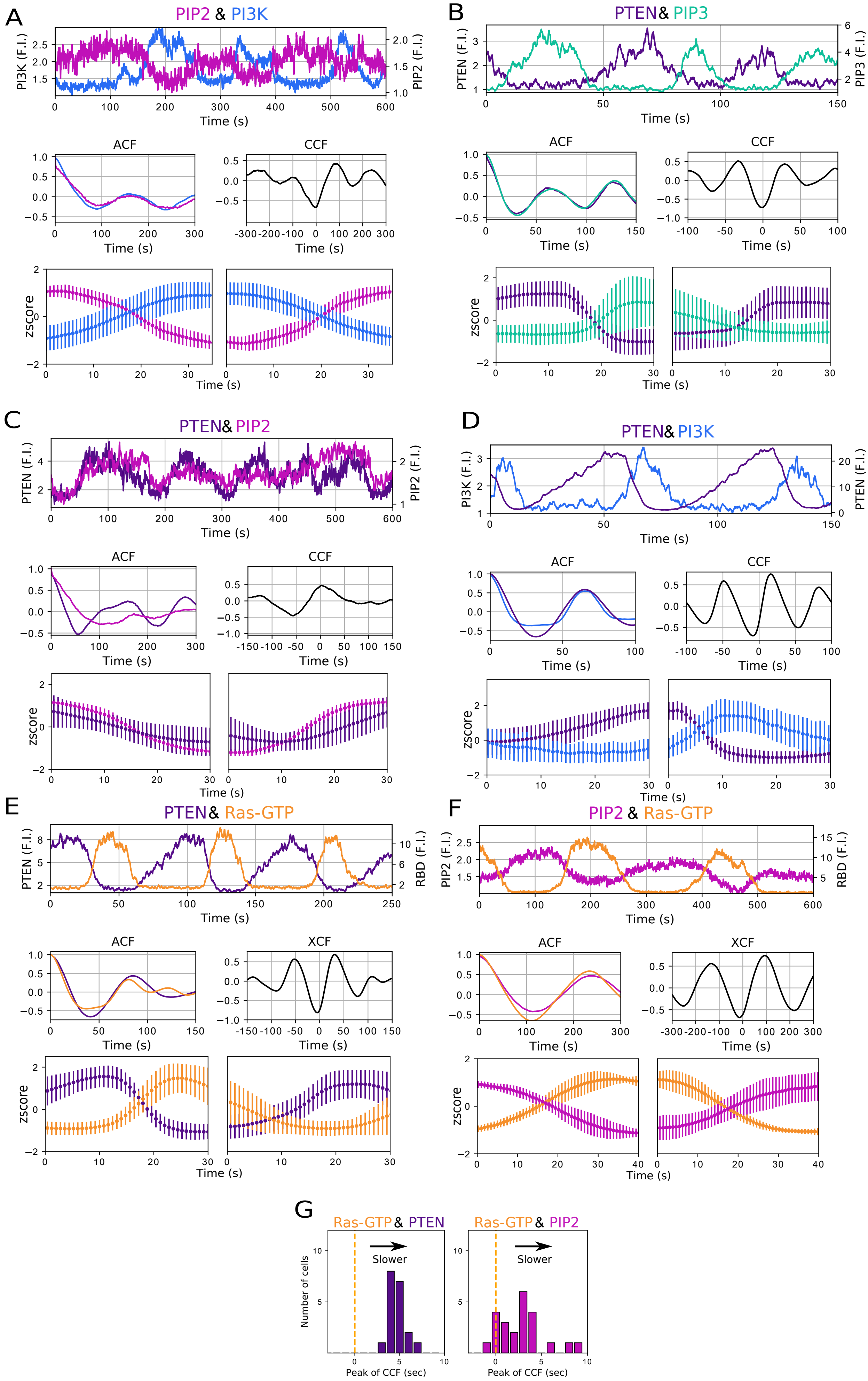
A
PIP2 & PI3K
PI3K (F.I.)
PIP2 (F.I.)
Time (s)
ACF
CCF
zscore
B
PTEN & PIP3
PTEN (F.I.)
PIP3 (F.I.)
C
PTEN & PIP2
D
PTEN & PI3K
E
PTEN & Ras-GTP
RBD (F.I.)
XCF
F
PIP2 & Ras-GTP
G
Ras-GTP & PTEN
Ras-GTP & PIP2
Slower
Number of cells
Peak of CCF (sec)

Fig. S4. Temporal relationship between Ras-GTP, PI3K, PTEN, PIP3 and PIP2. Typical time trajectories, auto- and cross-correlation functions and average trajectories of (A) PI3K2-Halo-TMR and GFP-Nodulin (n = 19 cells), (B) PTEN-Halo-TMR and $PHD_{AKT/PKB}$-GFP (n = 22 cells), (C) PTEN-Halo-TMR and GFP-Nodulin (n = 19 cells), (D) PI3K2-Halo-TMR and PTEN-GFP (n = 20 cells), (E) PTEN-Halo-TMR and RBD_{Raf1}-GFP (n = 19 cells) and (F) RFP-Nodulin and RBD_{Raf1}-GFP (n = 23 cells). (G) Distribution of peak times of the cross-correlation functions. Dotted lines indicate time zero. The average peak value of Ras-GTP against PTEN is 4.7 ± 0.8 s (n = 19 cells) and Ras-GTP against PIP2 is 2.7 ± 2.4 s (n = 23 cells).

Journal of Cell Science • Supplementary information

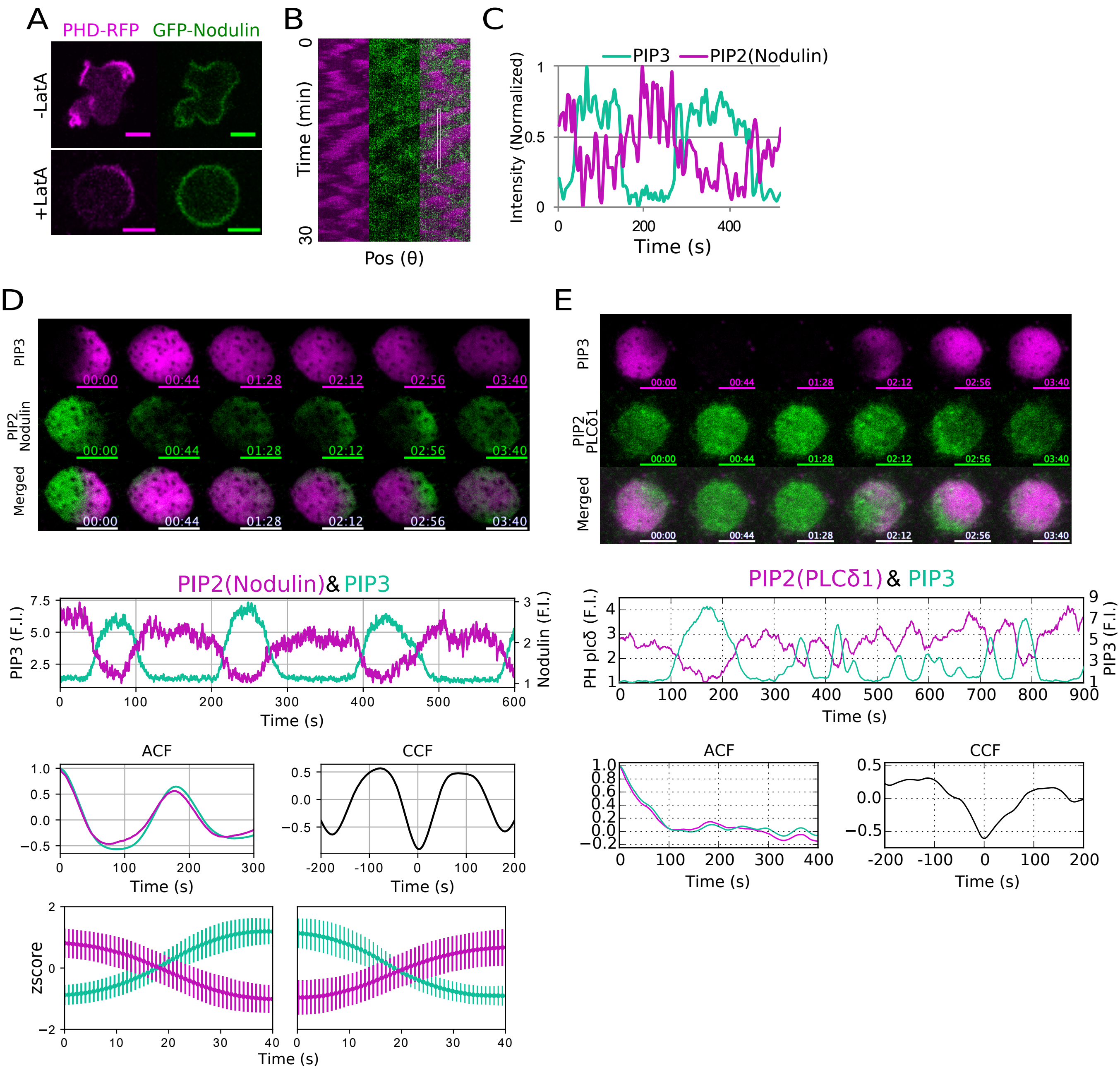

Fig. S5. Observation of PIP2 with Nodulin and PHD$_{PLC\delta1}$. (A) Confocal images of PIP3 (PHD$_{AKT/PKB}$-RFP) and GFP-Nodulin before and after treatment with Latrunculin A. (B) Kymographs of the cell periphery shown in Fig. S5A, bottom. (C) Time trajectory of PIP3 and PIP2 localization patterns in the ROI shown in the kymograph. (D), (E) Simultaneous time-lapse TIRFM imaging, typical time trajectories, auto- and cross-correlation functions of PIP3 and GFP-Nodulin and PHD$_{PLC\delta1}$-GFP. Scale bars, 5 μm. Time format is “mm:ss”. Average trajectories of simultaneous TIRF time-lapse imaging of PIP3 and GFP-Nodulin is also shown (bottom left, n = 26 cells).

Journal of Cell Science • Supplementary information

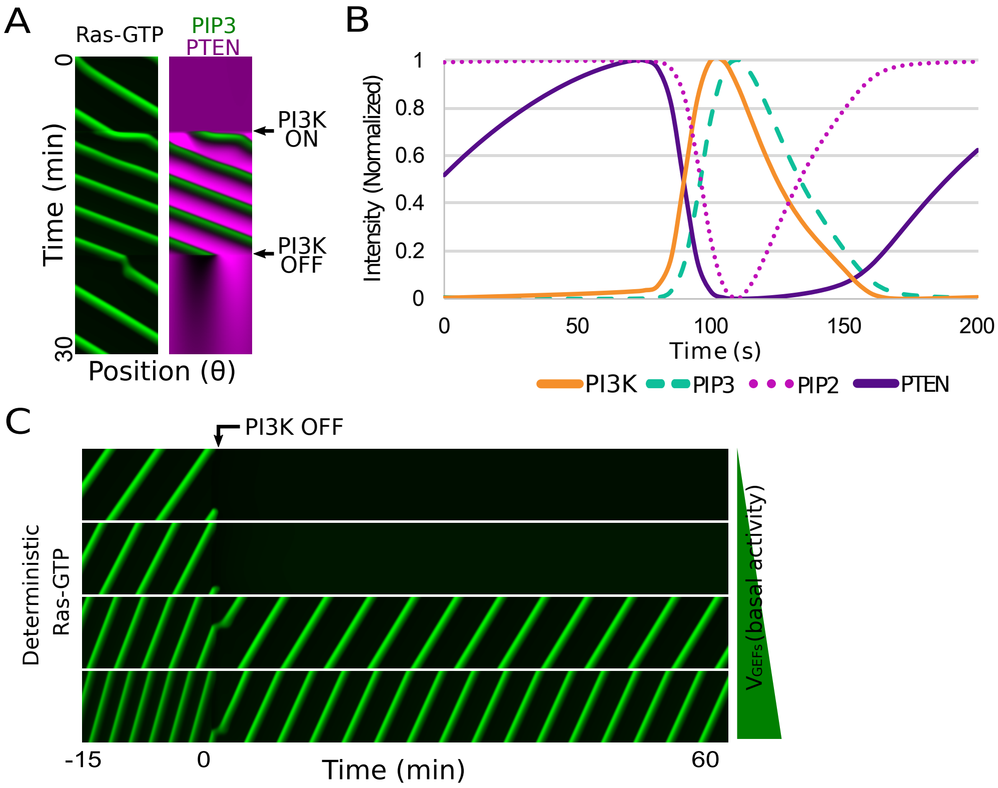

Fig S6. Deterministic simulation of Ras-GTP waves. (A) The kymograph generated by a simulation based on the model. V_{GEFs} = 550 s^{-1}. (B) Time trajectory of Ras-GTP/PI3K, PIP3, PIP2 and PTEN obtained from the simulation. (C) Simulation results of the PI3K inhibition experiment. The termination of Ras waves depended on the basal activity (V_{GEFs} = 450, 500, 550, 600 s^{-1}).

Table S1. Parameters for simulation (PIP3).

Parameters	Description	Values
V_{PTEN}	Dephosphorylation rate of PIP3 by PTEN	3 s^{-1}
K_{PTEN}	Michaelis constant of PTEN dephosphorylation reaction	500 μm^{-2}
V_{PI3K}	Phosphorylation rate of PIP2 by PI3K	12 s^{-1}
K_{PI3K}	Michaelis constant of PI3K phosphorylation reaction	300 μm^{-2}
V_{PTEN_ass}	PTEN association reaction rate by PIP2	5000 molecules$\mu M^{-1} \mu m^{-2} s^{-1}$
K_{PIP2}	Michaelis constant of PTEN association reaction	2000 μm^{-2}
λ_{PIP3}	PTEN-independent PIP3 degradation rate	0.2 s^{-1}
β	Parameter indicates magnitude of PI3K activation by Ras	0.01
χ	A constant to transform surface concentration to volume concentration	0.001 molecules$\mu M \mu m^{-2}$
$PTEN_{total}$	Total concentration of PTEN	0.1 μM
PIP_{total}	Density of PIPs on plasma membrane	5000 moleculesμm^{-2}
R	Cell radius	5 μm
D	Diffusion coefficient of molecules on plasma membrane	0.2 $\mu m^{-2} s^{-1}$
$[PIP3]_{initial}$	Initial value of [PIP3]	0 moleculesμm^{-2}
$[PIP2]_{initial}$	Initial value of [PIP2]	5000 moleculesμm^{-2}
$[PTEN]_{initial}$	Initial value of [PTEN]	0 moleculesμm^{-2}

Table S2. Parameters for simulation (Ras).

Parameters	Description	Values
V_{GAPs}	Deactivation rate of Ras by GAPs	16 s^{-1}
K_{GAPs}	Michaelis constant of GAPs deactivation reaction	40 moleculesμm^{-2}
V_{GEFs}	Activation rate of Ras by GEFs	400 ~ 600 s^{-1}
K_{GEFs}	Michaelis constant of GEFs activation reaction	3500 moleculesμm^{-2}
$V_{feedback}$	Reaction rate of PIP3 feedback regulation	25 s^{-1}
K_{PIP3}	Michaelis constant of PIP3 feedback regulation	0.1 moleculesμm^{-2}
λ_{RasGTP}	Dissociation rate of RasGTP	0.2 s^{-1}
λ_{RasGDP}	Dissociation rate of RasGDP	0.003 s^{-1}
k	Association rate of RasGDP	45 molecules$\mu m^{-2}s^{-1}$
V_{GAPs_ass}	Association rate of GAPs by RasGDP	1300 molecules$\mu m^{-2}\mu M^{-1}s^{-1}$
K_{RasGDP}	Michaelis constant of GAPs association reaction	3000 moleculesμm^{-2}
λ_{GAPs}	Dissociation rate of GAPs	1.2 s^{-1}
K_{α}	Half-maximum concentration of [RasGTP] for negative regulation of GAPs	120 moleculesμm^{-2}
α	Parameter indicates magnitude of negative regulation of GAPs	0.001
$GAPs_{total}$	Total concentration of GAPs	0.1 μM
$[RasGTP]_{initial}$	Initial value of [RasGTP]	1000 moleculesμm^{-2}
$[RasGDP]_{initial}$	Initial value of [RasGDP]	1000 moleculesμm^{-2}
$[GAPs]_{initial}$	Initial value of [GAPs]	0 moleculesμm^{-2}

Journal of Cell Science • Supplementary information

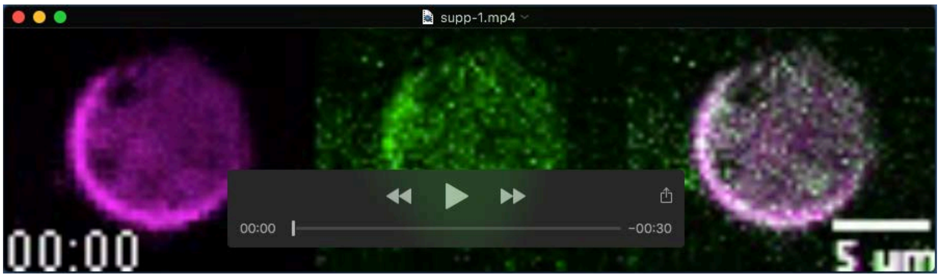

Movie S1. RBD$_{Raf1}$-RFP & PHD$_{AKT/PKB}$-GFP / Ax2. Time-lapse confocal images show RBD$_{Raf1}$-RFP (left), PHD$_{AKT/PKB}$-GFP (center) and merged (right), corresponding to Fig. 1A. Scale bars represent 5 µm. Time format is “mm:ss”.

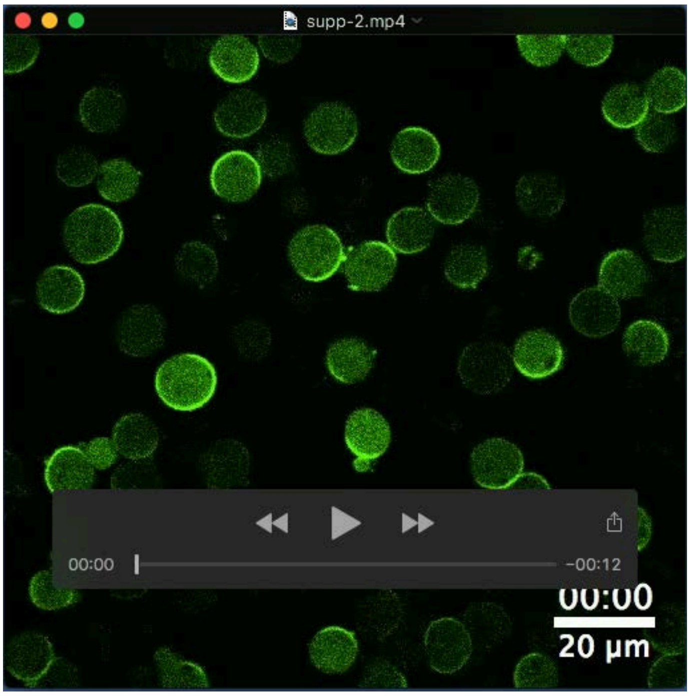

Movie S2. RBD$_{Raf1}$-RFP & PHD$_{AKT/PKB}$-GFP / Ax2 (TIRF). Time-lapse TIRF images show RBD$_{Raf1}$-RFP (left), PHD$_{AKT/PKB}$-GFP (center) and merged (right), corresponding to Fig. 2A. Scale bars represent 10 µm. Time format is “mm:ss”.

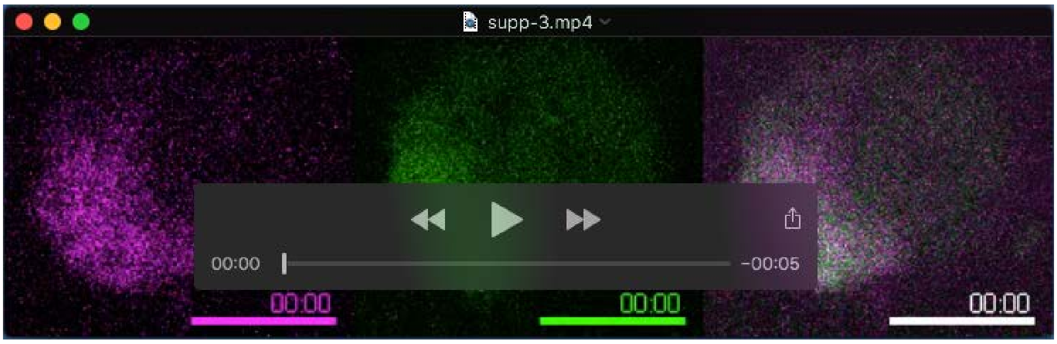

Movie S3. PI3K2-Halo-TMR & RBD$_{Raf1}$-GFP/Ax2 (TIRF). Time-lapse TIRF images show PI3K2-Halo-TMR (left), RBD$_{Raf1}$-GFP (center) and merged (right), corresponding to Fig. S2B.

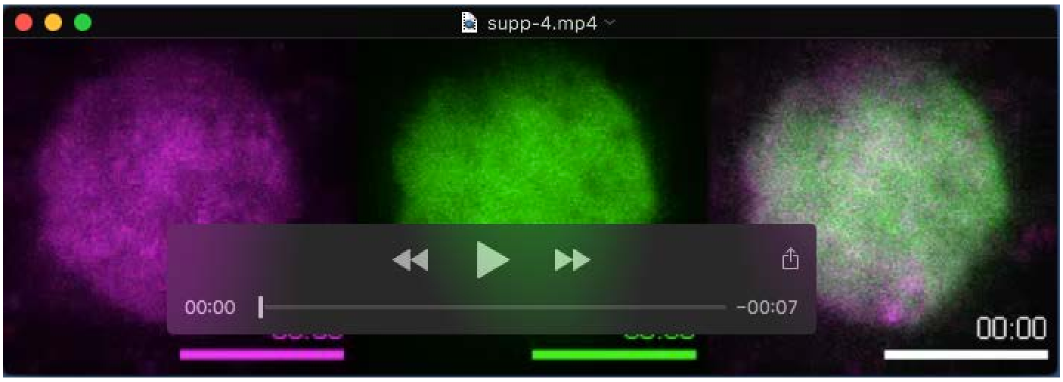

Movie S4. PI3K2-Halo-TMR & PHD$_{AKT/PKB}$-GFP/Ax2 (TIRF). Time-lapse TIRF images show PI3K2-Halo-TMR (left), PHD$_{AKT/PKB}$-GFP (center) and merged (right), corresponding to Fig. S2E.

Journal of Cell Science • Supplementary information

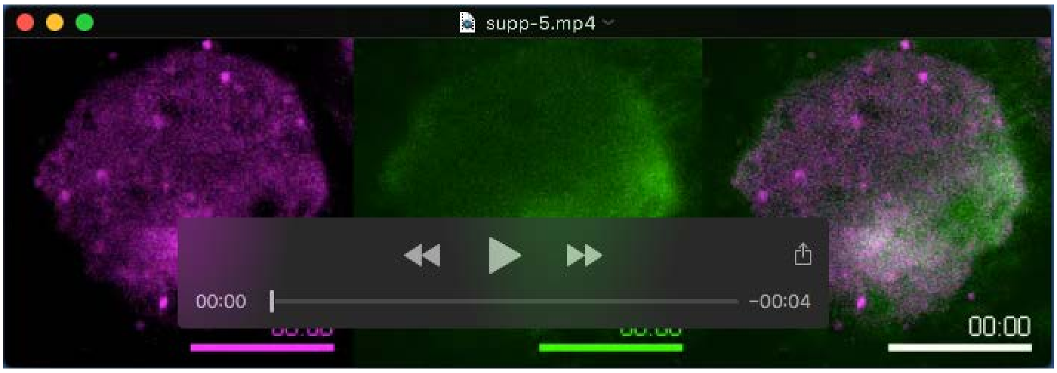

Movie S5. PI3K2(KE)-Halo-TMR & RBD$_{Raf1}$-GFP/Ax2 (TIRF). Time-lapse TIRF images show PI3K2$^{K857, 858E}$-Halo-TMR (left), RBD$_{Raf1}$-GFP (center) and merged (right), corresponding to Fig. 3A.

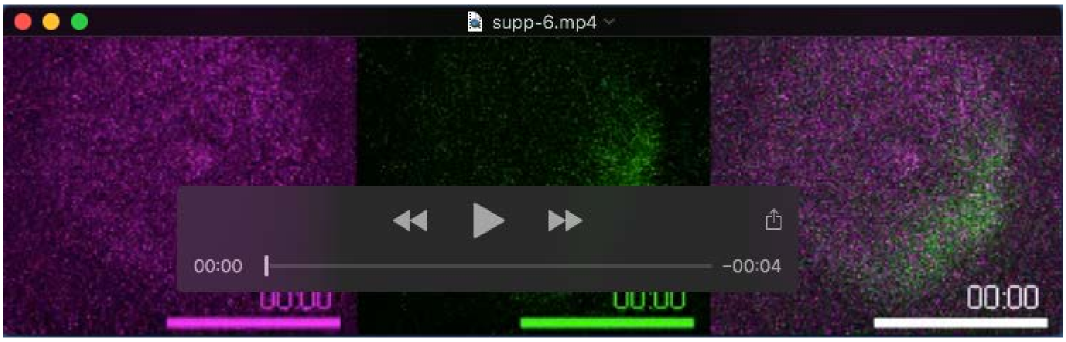

Movie S6. PHD$_{AKT/PKB}$-RFP & GFP-Nodulin/Ax2 (TIRF). Time-lapse TIRF images show PHD$_{AKT/PKB}$-RFP (left), GFP-Nodulin (center) and merged (right), corresponding to Fig. S5D.

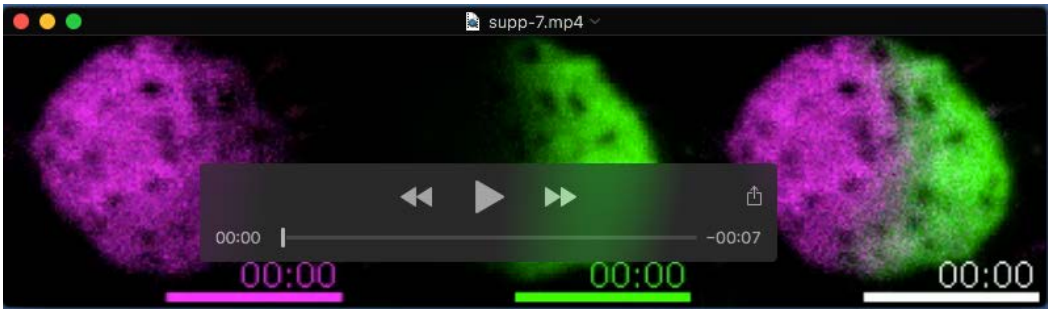

Movie S7. PTEN-Halo-TMR & PHD$_{AKT/PKB}$-GFP /Ax2, PTEN-Halo-TMR & GFP-Nodulin /Ax2, RFP-Nodulin & RBD$_{Raf1}$-GFP /Ax2, PI3K2-Halo-TMR & GFP-Nodulin /Ax2, PTEN-Halo-TMR & RBD$_{Raf1}$-GFP /Ax2, PI3K2-Halo-TMR & PTEN-GFP /Ax2 (TIRF). Time-lapse TIRF images show RFP or Halo-TMR conjugated proteins (left), GFP conjugated proteins (center) and merged (right), corresponding to S5 and Fig. S4.

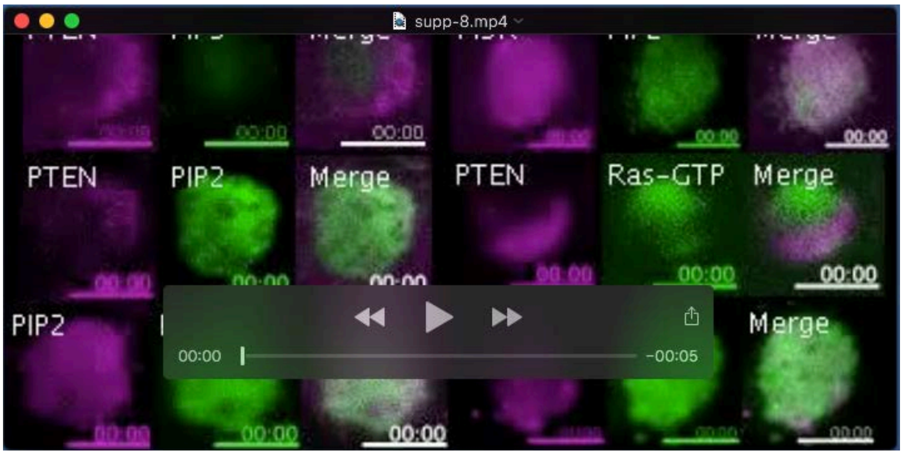

Movie S8. RBD$_{Raf1}$-GFP (+100 μM LY294002). Time-lapse confocal images show RBD$_{Raf1}$-GFP with 100 μM LY294002, corresponding to Fig. 4A. LY294002 was added at time 1 min. Scale bars represent 20 µm. Time format is “mm:ss”.
